# Pathways to, and use of, sexual healthcare among Black Caribbean sexual health clinic attendees in England: evidence from cross-sectional bio-behavioural surveys

**DOI:** 10.1186/s12913-019-4396-3

**Published:** 2019-09-18

**Authors:** Catherine R. H. Aicken, Sonali Wayal, Paula B. Blomquist, Stella M. Fabiane, Makeda Gerressu, Gwenda Hughes, Catherine H. Mercer

**Affiliations:** 1 Centre for Population Research in Sexual Health and HIV, Institute for Global Health, University College London (UCL), Mortimer Market Centre, London, WC1E 6JB UK; 2 HIV & STI Department, Centre for Infectious Disease Surveillance and Control, Public Health England (PHE), 61 Colindale Ave, London, NW9 5EQ UK; 3 National Institute for Health Research Health Protection Research Unit (NIHR HPRU) in Blood Borne and Sexually Transmitted Infections at UCL, in partnership with PHE, and in collaboration with the London School of Hygiene & Tropical Medicine, London, UK; 4 School of Health Sciences, University of Brighton, Village Way, Falmer, Brighton, BN1 9PH UK; 5 Instituto de Medicina Tropical, Universidade de São Paulo, São Paulo, Brazil

**Keywords:** Ethnicity, Black Caribbean, Sexually transmitted infections, Health inequalities, Sexual health clinics, Healthcare behaviour, Health behaviour

## Abstract

**Background:**

In England, people of Black Caribbean (BC) ethnicity are disproportionately affected by sexually transmitted infections (STI). We examined whether differences in sexual healthcare behaviours contribute to these inequalities.

**Methods:**

We purposively selected 16 sexual health clinics across England with high proportions of attendees of BC ethnicity. During May–September 2016, attendees at these clinics (of all ethnicities) completed an online survey that collected data on health service use and sexual behaviour. We individually linked these data to routinely-collected surveillance data. We then used multivariable logistic regression to compare reported behaviours among BC and White British/Irish (WBI) attendees (*n* = 627, *n* = 1411 respectively) separately for women and men, and to make comparisons by gender within these ethnic groups.

**Results:**

BC women’s sexual health clinic attendances were more commonly related to recent bacterial STI diagnoses, compared to WBI women’s attendances (adjusted odds ratio, AOR 3.54, 95% CI 1.45–8.64, *p* = 0.009; no gender difference among BC attendees), while BC men were more likely than WBI men (and BC women) to report attending because of a partner’s symptoms or diagnosis (AOR 1.82, 95% CI 1.14–2.90; AOR BC men compared with BC women: 4.36, 95% CI 1.42–13.34, *p* = 0.014). Among symptomatic attendees, BC women were less likely than WBI women to report care-seeking elsewhere before attending the sexual health clinic (AOR 0.60, 95% CI 0.38–0.97, *p* = 0.039). No ethnic differences, or gender differences among BC attendees, were observed in symptom duration, or reporting sex whilst symptomatic. Among those reporting previous diagnoses with or treatment for bacterial STI, no differences were observed in partner notification.

**Conclusions:**

Differences in STI diagnosis rates observed between BC and WBI ethnic groups were not explained by the few ethnic differences which we identified in sexual healthcare-seeking and use. As changes take place in service delivery, prompt clinic access must be maintained – and indeed facilitated – for those at greatest risk of STI, regardless of ethnicity.

**Electronic supplementary material:**

The online version of this article (10.1186/s12913-019-4396-3) contains supplementary material, which is available to authorized users.

## Background

In Britain, people of Black Caribbean (BC) ethnicity are disproportionately affected by sexually transmitted infections (STIs) [1], in the general population [2, 3] and in the higher-risk population [4] of sexual health clinic (SHC) attendees [5–8]. BC people attending SHCs are eight times more likely to be diagnosed with gonorrhoea than White British attendees, and almost six times as likely to be diagnosed with syphilis [8]. These health inequalities are not fully explained either at a population level by differences in sexual behaviour or broader contextual factors [1, 3], nor at an individual or partnership level by sexual behavioural and partnership differences among SHC attendees [9].

BC people comprise 1.1% of England and Wales’ population [10]. Migration to the UK from the Caribbean’s former British colonies was encouraged during the 1950s and 1960s. The UK’s BC communities have been established for several decades, and most of England and Wales’ BC population is UK-born [11]. Therefore barriers to healthcare faced by new migrants, e.g. unfamiliarity with the National Health Service (NHS) or language barriers, are likely to be relatively uncommon in the BC population. However, care-seeking and health behaviours may be influenced by sociocultural [12] and structural factors (including experience of racism) [13].

In the UK, specialist SHCs account for the majority of non-chlamydial STI diagnoses and management [14], providing services to patients on an open-access basis: with no requirement for a referral, and irrespective of where they live. Primary care services (e.g. general practice) provide variable, non-specialist sexual healthcare to local populations [15–17]. Rapid access to STI testing for those at risk of STI, and rapid treatment and partner notification support for those diagnosed, can prevent onward transmission of STIs and harms associated with long-term infection [18, 19]. The individual and public health effectiveness of STI services is influenced by whether they are used by those at risk of STI, how promptly they are used, and whether those infected notify partners and abstain from sex until treatment completion.

The sexual healthcare behaviour of people of BC ethnicity in the UK is under-researched: a recent systematic review found few studies which focus on this topic [1]. Nationally-representative data from Britain’s general population (collected 2010–12) show that BC men and women were more likely to report SHC attendance within the previous 5 years than White British men and women, after adjusting for confounders [3]. In a 2004–5 survey in SHCs across England, symptomatic BC men (but not women) experienced less provider-delay in accessing clinics than symptomatic White men did, but no other statistically-significant differences were observed in either care-seeking, or in sexual behaviour since seeking care [20]. Access to SHCs has changed since then: patients’ pathways to clinic shortened between 2004 and 5 and 2009 [17], but subsequently may have lengthened [21]. We conjectured that ethnic differences in pathways to, and use of, SHCs, might exist and contribute to explaining the high STI diagnosis rates among BC SHC attendees. To explore this, we compared sexual healthcare seeking and use (hereafter, ‘sexual healthcare behaviours’) among BC and White British/Irish (WBI) attendees (the ethnic majority). We also compared sexual healthcare behaviours by gender, within these ethnic groups.

## Methods

### Study design, study population and sampling

We developed a Bio-Behavioural Enhanced Surveillance Tool (BBEST) to explore factors influencing STI among key risk groups [22]. Development of the BBEST included formative qualitative research with BC people [23], stakeholder engagement (which continued throughout the study) and piloting [22]. We purposively-selected 16 SHCs across England with high proportions of BC attendees. Between May and September 2016, people attending these clinics (of all ethnicities) were invited to complete the BBEST online survey (Additional file 6), which they accessed on tablets provided at the clinics, or their own devices. Screening questions routed eligible attendees (aged ≥15 years old and sexually-active in the past year) to the full survey, which included questions about sexual health service use, symptoms, sexual behaviour whilst symptomatic, and partner notification.^1^ Survey data were individually linked, with participants’ consent, to an extract of data routinely collected by SHCs for national STI surveillance (GUMCAD STI Surveillance System), including STI diagnoses. We restricted our analyses to the 627 BC and 1411 WBI attendees (99.4%) who gave their gender as male or female. (We present findings for other ethnic groups in Additional files 1, 3, 4 and 5). These sample sizes gave us adequate statistical power (80%) to detect as statistically significant (at the 5% level) differences by ethnic group (BC vs. WBI) of, for example, 5% vs. 12% among men and 5% vs. 11% among women (i.e. for behaviours with low prevalence), and differences by ethnic group (BC vs. WBI) of, for example, 40% vs. 53% among men and 40% vs. 52% among women (i.e. for more prevalent behaviours). These power calculations also allow for a design effect of 1.2, reflecting how participants were clustered by clinic.

### Data analysis

Data were analysed using STATA v14, using survey commands to take account of the clustering of participants by clinic. We stratified the data by gender, and used logistic regression to obtain crude odds ratios (ORs) comparing BC and WBI participants. We then stratified by these two ethnic groups, and used logistic regression to compare participants by gender. We used multivariable logistic regression to account for possible confounders, separately for each comparison, using saturated models. Statistical significance was considered as *p* < 0.05 for all analyses.

In our analysis of reasons for SHC attendance, we examined reasons for attendance that participants reported in the survey, and used clinical data to identify participants with recent STI diagnoses (within the 6 weeks prior to their attendance at which the survey was completed) which we considered very probably related to clinic attendance.^2^

### Ethics

This study was approved by the NRES Committee South Central – Oxford C, ref.: 15/SC/0223.

## Results

### Sample characteristics

Table 1 presents participants’ characteristics, and comparisons by ethnic group and gender (findings for a wider range of ethnic groups are presented in Additional file 1). In this section and throughout the text of the Results, we first comment on ethnic differences among women, then among men, and finally, gender differences among BC participants.

**Table 1:** Sample characteristics, including comparisons by ethnicity and gender

	Women				Men				Comparisons by gender			
	Black Caribbean %	White British/ Irish %	OR (95% CI): BC attendees compared to WBI (referent)	p	Black Caribbean %	White British/ Irish %	OR (95% CI): BC attendees compared to WBI (referent)	p	Among Black Caribbean attendees		Among White British/Irish attendees	
									OR (95% CI): BC women as referent	p	OR (95% CI): WBI women as referent	p
*Denominator*	*420*	*838*			*207*	*573*						
Age (years)				0.864				0.048		0.040		0.004
15–24	45.2%	46.4%	–		35.3%	26.7%	–		–			
25+	54.8%	53.6%	1.05 (0.59–1.88)		64.7%	73.3%	0.67 (0.45–1.00)		1.52 (1.02–2.25)		2.38 (1.39–4.06)	
Median	26	25			27	28						
(IQR)	(22–31)	(21–30)			(22–34)	(24–33)						
Born outside the UK	23.0%	8.8%	3.08 (1.33–7.11)	0.012	20.6%	9.5%	2.48 (1.27–4.86)	0.012	0.87 (0.47–1.61)	0.629	1.08 (0.69–1.69)	0.725
Education (above GCSEs or equivalent)	77.6%	84.9%	0.62 (0.46–0.83)	0.004	68.8%	83.8%	0.43 (0.26–0.70)	0.003	0.64 (0.46–0.89)	0.012	0.92 (0.75–1.13)	0.410
In any form of employment	70.9%	71.8%	0.96 (0.67–1.37)	0.806	73.9%	83.1%	0.57 (0.38–0.88)	0.014	1.16 (0.78–1.74)	0.435	1.94 (1.28–2.93)	0.004
Sexual orientation				0.033				0.004		0.040		< 0.001
Heterosexual	94.3%	92.4%	–		87.7%	75.4%	–		–		–	
Bisexual/homosexual/rather not say^a,b^	5.7%	7.6%	0.74 (0.56–0.97)		12.3%	24.6%	0.43 (0.25–0.73)		2.29 (1.04–5.03)		3.95 (2.19–7.12)	
Number of sexual partners, last 12 months												
				0.012				0.109		< 0.001		< 0.001
1	48.6%	41.1%	–		15.7%	22.4%	–		–		–	
> 1	51.4%	58.9%	0.74 (0.59–0.93)		84.3%	77.6%	1.55 (0.90–2.69)		5.08 (2.88–8.97)		2.42 (1.86–3.15)	
				< 0.001				0.797		< 0.001		< 0.001
< 5	91.5%	82.7%	–		61.6%	60.5%	–		–		–	
5 or more	8.5%	17.3%	0.44 (0.31–0.63)		38.4%	39.5%	0.95 (0.64–1.42)		6.75 (4.08–11.17)		3.12 (2.40–4.04)	
Any new sexual partners, past 12 months	52.9%	68.6%	0.52 (0.40–0.66)	< 0.001	82.6%	82.7%	0.99 (0.64–1.53)	0.963	4.21 (2.37–7.48)	< 0.001	2.19 (1.70–2.84)	< 0.001
Current partnership(s)^d^												
Steady	62.6%	63.7%	0.95 (0.75–1.20)	0.659	47.3%	48.7%	0.94 (0.67–1.33)	0.719	0.54 (0.34–0.83)	0.010	0.54 (0.41–0.72)	< 0.001
Uncommitted regular	22.3%	23.3%	0.94 (0.82–1.10)	0.404	35.2%	21.5%	1.98 (1.44–2.72)	< 0.001	1.89 (1.13–3.17)	0.020	0.90 (0.68–1.19)	0.432
Casual	21.7%	21.4%	1.02 (0.76–1.36)	0.888	37.6%	41.5%	0.85 (0.58–1.25)	0.378	2.17 (1.23–3.82)	0.011	2.61 (2.06–3.30)	< 0.001
None	15.7%	18.7%	0.81 (0.58–1.13)	0.195	17.1%	17.0%	1.01 (0.69–1.49)	0.962	1.11 (0.76–1.62)	0.563	0.89 (0.61–1.29)	0.501
Condom use at last sex with most recent sexual partner^e^	31.9%	28.7%	1.16 (0.77–1.74)	0.442	35.1%	34.4%	1.03 (0.72–1.49)	0.850	1.16 (0.70–1.91)	0.538	1.30 (1.01–1.67)	0.040
Self-perceived risk of STI^f^				0.275				0.830		< 0.001		< 0.001
Considers self at risk of one or more STIs	41.7%	46.1%	–		71.0%	72.1%	–		–		–	
‘I don’t think I am at risk of getting any STI’	58.3%	53.9%	1.20 (0.85–1.68)		29.0%	27.9%	1.05 (0.64–1.74)		0.29 (0.17–0.50)		0.33 (0.22–0.51)	

Notes: Data in this table were reported by participants during the survey. Additiona file 1 provides detailed data on other ethnic groups

^a^Numbers answering ‘rather not say’ were small (21 women, 16 men), and were combined with participants identifying as homosexual or bisexual, for increased statistical power

^b^Instead of completing our survey, 15 BC men who have sex with men (MSM) and 85 WBI MSM completed another survey targeted at MSM and provided linked data for that survey, and so our survey slightly under-sampled MSM. This did not affect women participants

^c^Including opposite- and same-sex partners. Those reporting no sexual partners in the last 12 months were ineligible for the survey, reflecting the STI focus of our research programme

^d^‘Steady’: married, and/or committed but unmarried. ‘Uncommitted regular’: not in a committed relationship but have sex regularly. ‘Casual’: have sex but not regularly and/or one-off sex partner(s). Participants could select more than one current partnership(s) type

^e^Response option ‘we only had oral sex’ (selected by *n* = 85) was treated as missing, as this (pre-defined) response option was ambiguous, for the purpose of this analysis (i.e. oral sex on a man can be with or without a condom)

^f^Based on responses to the question: ‘Thinking about your current sexual lifestyle, which of the following STIs do you think you may be at risk of?’ Response options comprised a list of STIs including HIV, and a ‘no risk’ response option, as provided in the table

BC and WBI women were similar in age (medians 26, 25 years respectively). 23.0% of BC and 8.8% of WBI women were born outside the UK. BC women were less likely than WBI women to be educated beyond GCSEs (77.6, 84.9%, respectively), but equally likely to be in work (just over 70%). Slightly more BC women than WBI women self-identified as heterosexual (94.3, 92.4% respectively). BC women reported fewer partners than WBI women: e.g. 8.5% BC women, compared with 17.3% WBI women, reported 5 or more partners within the past year. BC women were also less likely to report new partners in this timeframe. However, there were no ethnic differences in women’s current partnership type(s). There were also no ethnic differences in the proportions of women: reporting condomless last sex (around 70%), or considering themselves at risk of STI (around 44%).

Over a third (35.3%) of BC men were aged under 25, compared with 26.7% WBI men. 20.6% of BC men were born outside the UK, compared with 9.5% WBI men. A lower proportion of BC men than WBI men were educated beyond GCSEs (68.8, 83.8% respectively), and a lower proportion were in work (73.9, 83.1%). In our sample, 87.7% BC men self-defined as heterosexual, compared with 75.4% WBI men.^3^ No ethnic differences were observed in the number of partners men had in the past year; around 39% reported 5 or more partners, and almost 83% reported new partners. While there were no ethnic differences in the proportions of men reporting no current partnership, current steady or casual partnerships, 35.2% BC men reported uncommitted regular partnerships, compared with 21.5% WBI men. There were no ethnic differences in the proportions of men reporting condomless last sex (around 65%), nor in the proportions considering themselves at risk of STI (just over 70%).

BC men were somewhat older than BC women (OR for being age 25 or older: 1.52, 95% CI: 1.02–2.25). While there were no gender differences observed in the proportions of BC attendees born outside the UK, nor the proportions in employment, a lower proportion of BC men than BC women were educated beyond GCSEs (OR 0.64, 95% CI:0.46–0.89). Despite our survey slightly under-sampling MSM,^2^ BC men were more likely than BC women to self-identify as non-heterosexual (OR 2.29, 95%CI:1.04–5.03). BC men reported more partners in the past 12 months than BC women (OR for > 1 partner, vs. 1 partner: 5.08, 95%CI:2.88–8.97; OR for ≥5 partners, vs. < 5 partners: 6.75, 95% CI:4.08–11.17), and were more likely to report new partner(s) (OR 4.21, 95% CI:2.37–7.48). Although there were no gender differences among BC attendees in the proportions reporting no current partnerships, there were differences in current partnership types: a lower proportion of BC men than BC women reported being in steady partnership(s) (OR: 0.54, 95%CI:0.34–0.83), while a higher proportion reported uncommitted regular, or casual, partnership(s) (ORs: uncommitted regular: 1.89, 95% CI:1.13–3.17; casual: 2.17, 95% CI:1.23–3.82). There was no gender difference in reporting condomless last sex, but BC men were more likely than BC women to consider themselves at risk of STI (OR for considering not at risk of STI: 0.29, 95% CI:0.17–0.50).

### Reasons for attending SHCs

**Fig. 1:**
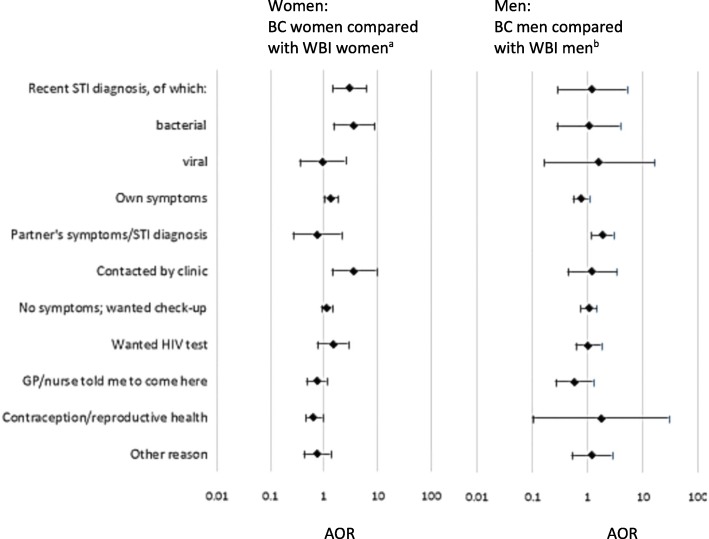
Comparison of reasons for attendance, by ethnic group. Multiple reasons could apply. Adjusted Odds Ratios, adjusted for variables which were statistically significant at *p* < 0.05 in Table 1. ^a^For the ethnic comparison among women: the following binary variables: born in UK, education, sexual orientation, > 5/5+ partners in past 12 months (other sexual partner number variables were omitted due to likely covariance). ^b^For the ethnic comparison among men: age as a continuous variable, and the following binary variables: born in UK, education, employment, sexual orientation, reporting regular but uncommitted partner(s). Additional file 2 provides the data used in Fig. 1 including within ethnic group comparisons by gender, and Additional file 3 provides detailed data on other ethnic groups

The two main reasons for attending clinic were having (had) symptoms, and wanting an asymptomatic check-up, together reported by almost three-quarters of attendees. After adjusting for variables which were statistically significant in Table 1, BC women’s attendance was more likely to be related to recent STI diagnosis/es, than WBI women’s, and specifically bacterial STI diagnosis/es (AOR 2.98, 95% CI:1.43–6.23; 4.1% BC and 1.3% WBI women had recent bacterial STI diagnosis/es). BC women were more likely than WBI women to report attending because they were contacted by the clinic (AOR 3.64, 95% CI:1.41–9.38), but less likely to report attending for a contraceptive or reproductive health reason (AOR 0.64, 95% CI:0.45–0.92). No other ethnic differences were observed in women’s reasons for attendance (Fig. 1, data presented in Additional file 2). (Additional file 3 presents findings for a wider range of ethnic groups).

Among men, no statistically-significant ethnic differences were observed in the proportions with recent STI diagnosis/es, nor in reported reasons for attendance, except that BC men were more likely to report attending because of a *partner’s* symptoms or STI diagnosis (AOR 1.81, 95%CI:1.18–2.79).

Among BC attendees, no gender differences were observed in recent STI diagnoses. The only reasons for attendance which differed by gender were: partner’s symptoms/diagnosis (more commonly-reported by BC men than BC women, AOR: 4.36, 95% CI:1.42–13.34); and contraceptive/reproductive health reasons (unsurprisingly much less commonly-reported by BC men than BC women, AOR 0.04, 95% CI:< 0.01–0.70).

### Symptomatic attendees’ pathways to clinic

We now focus on the pathways to clinic of participants who reported being symptomatic as a reason for attendance, around four-in-ten of the sample (Table 2; Additional file 4 presents findings for a wider range of ethnic groups).

**Table 2:** Pathways to sexual health clinics by ethnic group and gender, among those reporting symptoms

	Women (WBI women as reference category)				Men (WBI men as reference category)			Comparisons by gender				
	Black Caribbean %	White British/Irish %	OR (95% CI), p	AOR^a^ (95% CI), p	Black Caribbean %	White British/Irish %	OR (95% CI), p	AOR^b^ (95% CI), p	Among BC attendees (BC women as reference category)		Among WBI attendees (WBI women as ref. category)	
									OR (95% CI), p	AOR^c^ (95% CI), p	OR (95% CI), p	AOR^d^ (95% CI), p
*Denominator*	*169*	*296*			*81*	*267*						
Time since symptoms started			*p* = 0.392	*p* = 0.372			*p* = 0.287	*p* = 0.337	*p* = 0.295	*p* = 0.657	*p* = 0.584	*p* = 0.813
> 7 days ago	63.1%	67.2%	–	–	55.6%	65.4%	–	–	–	–	–	–
≤ 7 days	36.9%	32.8%	1.20 (0.77–1.86)	1.21 (0.78–1.86)	44.4%	34.6%	1.51 (0.68–3.38)	1.51 (0.62–3.66)	1.37 (0.74–2.53)	1.25 (0.43–3.61)	1.09 (0.80–1.48)	0.94 (0.54–1.63)
Sought treatment/ advice for symptoms elsewhere, before attending study clinic	35.1%	45.2%	*p* = 0.099 0.66 (0.39–1.09)	p = 0.039 0.60 (0.38–0.97)	30.0%	35.3%	*p* = 0.309 0.78 (0.48–1.28)	*p* = 0.279 0.75 (0.43–1.30)	*p* = 0.257 0.79 (0.52–1.21)	*p* = 0.337 0.68 (0.29–1.57)	*p* = 0.024 0.66 (0.47–0.94)	*p* = 0.092 0.80 (0.61–1.04)
Had sex since symptoms started			*p* = 0.041	*p* = 0.068			*p* = 0.904	*p* = 0.974	*p* = 0.347	*p* = 0.816	*p* = 0.005	*p* = 0.003
No	57.5%	47.5%	–	–	61.7%	62.6%	–	–	–	–	–	–
Yes, with one partner	40.1%	45.4%			28.4%	31.7%						
			0.67 (0.46–0.98)	0.67 (0.43–1.04)			1.04 (0.53–2.06)	0.99 (0.44–2.20)	0.84 (0.57–1.24)	1.07 (0.60–1.91)	0.54 (0.36–0.81)	0.53 (0.36–0.78)
Yes, with > 1 partner	2.4%	7.1%			9.9%	5.7%						

Notes: Data in this table were reported by participants during the survey, and denominators are participants reporting symptoms as a reason for clinic attendance. We assume that those with STI symptoms will have reported these symptoms as a reason for attendance. Additional file 4 provides detailed data on other ethnic groups

^a,b,c,d^Adjusted Odds Ratios are adjusted for variables which were statistically significant at *p* < 0.05 in Table 1:

^a^For the ethnic comparison among women: the following binary variables: born in UK, education, sexual orientation, > 5/5+ partners in past 12 months (other sexual partner number variables were omitted due to likely covariance)

^b^For the ethnic comparison among men: age as a continuous variable, and the following binary variables: born in UK, education, employment, sexual orientation, reporting regular but uncommitted partner(s)

^c^For the gender comparison among BC attendees: age as a continuous variable, and the following binary variables: education, sexual orientation, > 5/5+ partners in past 12 months (other sexual partner number variables were omitted due to likely covariance), reporting any steady partner(s), reporting any regular but uncommitted partner(s), reporting any casual partner(s), and self-perceived STI risk

^d^For the gender comparison among White British/Irish attendees: age as a continuous variable, and the following binary variables: employment, sexual orientation, > 5/5+ partners in past 12 months (other sexual partner number variables were omitted due to likely covariance), reporting any steady partner(s), reporting any casual partner(s), reporting condom use at last sex, and self-perceived STI risk

Around a third of symptomatic women reported symptom onset within the last 7 days, and around half reported having had sex since symptoms began, with no ethnic differences observed. Symptomatic BC women were less likely than their WBI counterparts to report having tried to get treatment/advice elsewhere before attending clinic (AOR: 0.60, 95% CI:0.38–0.97). No ethnic differences were observed among symptomatic men, in reporting: duration of symptoms, seeking treatment/advice elsewhere before attending clinic, or sex since symptoms began. No gender differences in these variables were observed among symptomatic BC attendees.

### Attendees’ previous experiences of STI and partner notification

We now focus on the subsample of attendees reporting previous STI diagnosis or treatment (hereafter ‘previous STI’), exploring their most recent episode (Table 3; Additional file 5 presents findings for a wider range of ethnic groups). Previous STI was more commonly reported by BC women than WBI women (61.2% compared with 40.0%), with a smaller difference between BC and WBI men (60.9, 50.6% respectively), and no gender difference among BC attendees.

**Table 3:** Previous experience of STI and partner notification

	Women (WBI women as reference category)				Men (WBI men as reference category)				Comparisons by gender			
	Black Caribbean %	White British/Irish %	OR (95% CI), p	AOR^f^ (95% CI), p	Black Caribbean %	White British/Irish %	OR (95% CI), p	AOR^g^ (95% CI), p	among BC attendees (BC women as referents)		among WBI attendees (WBI women as referents)	
									OR (95% CI), p	AOR^h^ (95% CI), p	OR (95% CI), p	AOR^i^ (95% CI), p
Of all participants												
*Denominator*	*420*	*838*			*207*	*573*						
Ever before diagnosed with (or treated for) an STI^**a**^	61.2%	40.0%	*p* < 0.001 2.37 (1.93–2.91)	*p* < 0.001 2.54 (1.98–3.27)	60.9%	50.6%	*p* = 0.016 1.52 (1.10–2.10)	*p* = 0.006 1.67 (1.19–2.33)	*p* = 0.923 0.99 (0.73–1.33)	*p* = 0.025 0.57 (0.36–0.92)	*p* = 0.005 1.54 (1.17–2.03)	*p* = 0.063 1.29 (0.98–1.69)
Of those reporting previous STI diagnosis/treatment^**a**^												
*Denominator*	*257*	*335*			*126*	*290*						
Last time this happened			*p* = 0.314	*p* = 0.331			*p* = 0.329	*p* = 0.647	p = 0.003	*p* = 0.226	*p* = 0.735	*p* = 0.055
More than 12 months ago	61.5%	55.5%	*–*	–	49.2%	53.4%	–	–	–	–	–	–
Within the last 12 months	38.5%	44.5%	0.78 (0.47–1.30)	0.80 (0.49–1.29)	50.8%	46.6%	1.19 (0.83–1.70)	1.10 (0.70–1.74)	1.65 (1.22–2.22)	1.35 (0.81–2.22)	1.09 (0.65–1.83)	1.32 (0.99–1.74)
Last STI diagnosed/treated was a bacterial STI or trichomoniasis	71.6%	60.0%	*p* < 0.001 1.68 (1.39–2.03)	*p* < 0.001 1.97 (1.52–2.55)	74.6%	54.1%	*p* = 0.002 2.49 (1.50–4.12)	*p* < 0.001 3.37 (2.17–5.24)	*p* = 0.481 1.17 (0.74–1.83)	*p* = 0.675 1.17 (0.54–2.55)	*p* = 0.177 0.79 (0.55–1.13)	*p* = 0.029 0.57 (0.35–0.94)
Of those reporting bacterial STI(s) or trichomoniasis,^c^ at last STI episode												
*Denominator*	*184*	*201*			*94*	*157*						
At that time, did the clinic staff advise you to inform your sexual partners to test for STIs/come to clinic?			*p* = 0.912	*p* = 0.941			*p* = 0.274	*p* = 0.207	*p* = 0.485	*p* = 0.369	*p* = 0.221	*p* = 0.230
No	8.7%	9.5%	} *-*	–	6.4%	10.8%	} *-*	–	–	–		
Can’t remember	7.1%	7.0%	}		12.8%	15.3%	}					
Yes	84.2%	83.6%	1.05 (0.42–2.65)	1.04 (0.38–2.82)	80.9%	73.9%	1.49 (0.70–3.17)	1.59 (1.75–3.37)	0.79 (0.39–1.60)	0.60 (0.18–1.97)	0.56 (0.21–1.49)	0.53 (0.18–1.57)
At that time, did you inform your sexual partners to test/take treatment for STIs?			*p* = 0.123	*p* = 0.767			*p* = 0.008	p = 0.006	*p* = 0.705	*p* = 0.654	*p* = 0.001	*p* = 0.020
No, I didn’t tell any partners	6.0%	10.9%	} 0.64	0.77	8.5%	20.4%	} 0.28	0.30	1.15	0.77	2.62	2.12
Can’t remember	7.1%	8.5%	} (0.36- } 1.15)	(0.37–1.58)	8.5%	8.3%	} (0.12 } 0.68)	(0.14–0.67)	(0.54–2.44)	(0.22–2.69)	(1.56–4.40)	(1.15–3.94)
Yes, I told SOME of my partners	6.5%	7.0%	}		5.3%	15.3%	}					
Yes, I told ALL my partners	80.4%	73.6%	*–*	–	77.7%	56.1%	–	–	–	–	–	–
Of those reporting being diagnosed with (or receiving treatment for) bacterial STI(s)/ trichomoniasis,^c^ at last STI episode AND who informed some/all partners												
*Denominator*	*160*	*162*			*78*	*112*						
At that time, how did you inform you sexual partners to test for STIs/come to clinic?^b^												
In person	67.7%	57.9%	*p* = 0.184 1.53 (0.80–2.93)	*p* = 0.229 1.42 (0.78–2.58)	65.4%	59.5%	*p* = 0.442 1.29 (0.65–2.56)	*p* = 0.613 0.83 (0.38–1.81)	*p* = 0.706 0.90 (0.50–1.62)	*p* = 0.831 0.91 (0.34–2.40)	*p* = 0.810 1.07 (0.60–1.90)	*p* = 0.060 1.81 (0.97–3.37)
Via text message/email/ phone/social media	44.9%	54.1%	*p* = 0.013 0.69 (0.53–0.91)	*p* = 0.108 0.81 (0.62–1.05)	48.7%	57.7%	*p* = 0.140 0.70 (0.43–1.14)	*p* = 0.863 1.06 (0.51–2.22)	*p* = 0.415 1.16 (0.79–1.72)	*p* = 0.837 0.95 (0.58–1.56)	*p* = 0.559 1.16 (0.69–1.94)	*p* = 0.162 0.56 (0.24–1.30)
Via a clinic health adviser/clinic staff	3.2%	2.5%	*p* = 0.729 1.27 (0.30–5.31)	*p* = 0.708 1.31 (0.29–6.00)	1.3%	0.9%	*p* = 0.823 1.43 (0.05–41.7)	*p* = 0.183 8.48 (0.32–222.8)	*p* = 0.425 0.40 (0.04–4.42)	p = 0.183 0.29 (0.04–1.92)	*p* = 0.406 0.35 (0.03–4.79)	*p* = 0.197 0.24 (0.03–2.29)
Other	0.6%	1.3%	*p* = 0.322 0.50 (0.11–2.19)	*p* = 0.377 0.53 (0.12–2.36)	2.6%	1.8%	*p* = 0.663 1.43 (0.25–8.23)	p = 0.008 8.49 (1.91–37.75)	*p* = 0.261 4.13 (0.31–55.42)	*p* = 0.050 6.20 (1.00–38.46)	*p* = 0.716 1.44 (0.17–11.89)	*p* = 0.586 1.64 (0.25–11.83)
Of those reporting being diagnosed with (or receiving treatment for) bacterial STI(s)/trichomoniasis,^c^ at last STI episode AND who informed SOME/NO partners												
*Denominator*	Black Caribbean women *23*			White British/Irish women *36*			Black Caribbean men *13*			White British/Irish men *56*		
At that time, why did you not inform (some of) your sexual partners to test for the infection/come to the clinic?^b, e^ *(Top 3 most commonly selected reasons)*	‘I was embarrassed to tell my partner(s) about the infection’			‘I did not have contact details of my partner(s)’			‘I was embarrassed to tell my partner(s) about the infection’			‘I did not have contact details of my partner(s)’		
	‘I was scared of telling my partner(s) about the infection’			‘I was not too concerned about telling my casual / one-off partner(s)’			‘I was worried that my partner(s) would leave me’			‘I was embarrassed to tell my partner(s) about the infection’		
	[Joint 3rd most common responses:] ‘I did not have contact details of my partner(s)’			‘I was embarrassed to tell my partner(s) about the infection’			‘I was scared of telling my partner(s) about the infection’			‘I was not too concerned about telling my casual / one-off partner(s)’		
	‘I was not too concerned about telling my casual / one-off partner(s)’											

Notes: Data in this table were reported by participants during the survey. Additional file 5 provides detailed data on other ethnic groups

^a^STIs listed included: chlamydia, gonorrhoea, genital warts (venereal warts), syphilis, *Trichomonas vaginalis* (Trich, TV), herpes (genital herpes), hepatitis B, NSU/NGU (non-specific urethritis/non-gonococcal urethritis), epididymitis, HIV. Only a small minority - 61 women and 40 men - reported having been diagnosed within the last 7 days (i.e. perhaps no opportunity for PN yet)

^b^Multiple responses were permitted

^c^Chlamydia, gonorrhoea, syphilis, trichomoniasis, NSU/NGU, epididymitis. We only included bacterial STIs and trichomoniasis in the questions about partner notification (PN) because they are acute infections for which PN should be addressed when diagnosed/treated. (Viral STIs may be chronic and require repeat treatments, and therefore there would not necessarily have been a routine PN discussion when last treated; furthermore, PN is not routinely advised for some viral STIs, e.g. warts, herpes) [24].

^d^Including: via a friend, partner already knew, had already been told, or was tested at the same time; negative result/no STI (e.g. after waiting for results of testing for NGU); annoyance/anger at partner; no (other) partners to tell; used condom/protection with partner; wasn’t told to tell anyone; complicated nature of relationships with partners; can’t remember; didn’t feel the need to; etc.

^e^See Additional file 6 for full list of response options

^f,g,h,i^Adjusted Odds Ratios are adjusted for variables which were statistically significant at *p* < 0.05 in Table 1:

^f^For the ethnic comparison among women: the following binary variables: born in UK, education, sexual orientation, > 5/5+ partners in past 12 months (other sexual partner number variables were omitted due to likely covariance)

^g^For the ethnic comparison among men: age as a continuous variable, and the following binary variables: born in UK, education, employment, sexual orientation, reporting regular but uncommitted partner(s)

^h^For the gender comparison among BC attendees: age as a continuous variable, and the following binary variables: education, sexual orientation, > 5/5+ partners in past 12 months (other sexual partner number variables were omitted due to likely covariance), reporting any steady partner(s), reporting any regular but uncommitted partner(s), reporting any casual partner(s), and self-perceived STI risk

^i^For the gender comparison among White British/Irish attendees: age as a continuous variable, and the following binary variables: employment, sexual orientation, > 5/5+ partners in past 12 months (other sexual partner number variables were omitted due to likely covariance), reporting any steady partner(s), reporting any casual partner(s), reporting condom use at last sex, and self-perceived STI risk

Almost half of attendees reporting previous STI were last diagnosed or treated within the past year, with no differences by gender or ethnic group. BC women and BC men were more likely than their WBI counterparts to report that their most recent episode included bacterial STI(s) and/or TV (AORs: women: 1.97, 95% CI:1.52–2.55; men: 3.37, 95% CI:2.17–5.24). No statistically-significant gender differences were observed among BC attendees.

Of those reporting that their most recent STI was bacterial or TV, four-fifths recalled that clinic staff advised partner notification (PN), with no ethnic or gender differences. No ethnic differences were observed in the proportion of women reporting notifying all their partners (around four-fifths). However, BC men were more likely than WBI men to report notifying all of their partners (AOR for not notifying all partners, or cannot remember: 0.30, 95%CI:0.14–0.67). No gender differences in reporting this were observed among BC attendees.

Among those reporting notifying any partner(s) at this time, two-thirds reported notifying partner(s) in person, and half reporting doing so by telephone, text message, email, or social media (combined). Less than 4% had clinic staff notify partners for them. No ethnic or gender differences in notification methods were observed.

The three most commonly-reported reasons for not notifying (all) partners were similar between ethnic groups (however denominators were small). BC attendees of both genders reported being scared to tell partners (and BC men, being worried that their partner would leave them), which did not feature in WBI attendees’ top-3 reasons.

## Discussion

### Main findings

Our study showed that differences in STI diagnosis rates observed between BC and WBI ethnic groups were not explained by the few ethnic differences that were identified in sexual healthcare-seeking. These differences were that BC women were more likely than WBI women to have had recent bacterial STI diagnosis/es, or to attend because they were contacted by the clinic, while BC men were more likely than WBI men to report attendance due to a partner’s diagnosis. Symptom duration was similar between ethnic groups, but symptomatic BC women were less likely than symptomatic WBI women to report first seeking care elsewhere. We observed no ethnic differences in symptomatic attendees’ likelihood of reporting sex since symptom onset. Among those who had previously been diagnosed/treated for bacterial STI(s) or TV, there were no ethnic differences in reporting having been *advised* to notify partners, but BC men were more likely than WBI men to report notifying all of their partners at this time.

### Strengths and limitations

Our research follows recommendations to assess needs and inequalities by ethnicity, to guide practical action [25], and contributes findings about an epidemiologically-important population. Purposively-selecting clinics with high proportions of BC attendees enabled us to recruit a sample that included a relatively large number of this minority group. This enabled us to perform adjusted analyses (which were not done in a previous, similar study [20]) to control for observed differences in sociodemographic and sexual behavioural factors, which could otherwise obscure associations with health (care) behaviours. As others have done, we made gender-stratified comparisons by ethnicity [20], but additionally conducted ethnicity-stratified comparisons by gender, to explore the ‘effect’ of gender within ethnic groups.

Our findings are derived from detailed data collected at SHCs across England. Linkage to clinical data (achieved for the majority of survey-completers [22]) enabled inclusion of clinic-verified STI diagnoses. This has been done in previous studies of patients’ pathways to clinic [17, 26, 27], but we were able to link to longitudinal clinical data, to include STI diagnoses associated with earlier or later attendances at the same clinic. While it is not possible to link diagnoses made in different clinics (because patient identifiers are clinic-specific), our use of a narrow (+/− 6 weeks) ‘window’ for diagnoses data probably minimises the impact of this issue.

By surveying SHC attenders, we focus on a high STI risk population [4], with a key role in STI transmission and control. However, we were unable to collect data on those who did not use SHCs, and so do not know about non-attenders care-seeking or use of other services. In nationally-representative survey data, over 85% people who did not attend SHC in the past year, reported unsafe sex during this period [28]. Non-attenders also reported fewer markers of STI risk than attendees (after age-adjustment) [28], but nevertheless include people who are underserved, and thus epidemiologically-important.

Our analyses of sub-samples (e.g. Table 3) may have lacked the statistical power to detect some differences as significant, reflecting how we sought to examine ethnic differences within gender, and gender differences within ethnic group, which our original power calculations did not factor in. Our sampling strategy was designed to obtain sufficient numbers of SHC attendees of BC ethnicity, a relatively under-researched group. It was not designed to give us sufficient statistical power to additionally stratify our analyses by sexual orientation, and so we were unable to explore reasons why differences exist by sexual orientation, although these have been the focus of other studies [15, 28, 29]. Survey response rates were generally high (averaging 62.2%), but varied by clinic, related to clinics’ ability to support survey administration [22]. Among survey-completers, consent to data linkage was slightly lower among BC than White participants (87.1% vs. 93.8%, *p* < 0.01) [22]. This is still relatively high, which minimises any differential influence on representativeness by ethnicity. We found some evidence that we may have over-sampled those with higher risk-profiles than SHC attendees in general [22]. The effect on our findings is unknown, but the highest-risk individuals are those of greatest concern in terms of individual and public health need.

All self-reported data is potentially subject to social desirability bias, but confidential, electronic self-completion may reduce this [30, 31]. Sexual health knowledge may differ by ethnicity (suggested by large survey of London young people [32]), which could influence actual and reported behaviours, and ethnic differences therein.

### Discussion of findings in relation to other research

Our sample of BC SHC attendees is likely to be at higher STI risk than the BC population as a whole [33], or the general population [4]. We found ethnic differences in reasons for attendance, whereas no differences were observed in a 2004–05 clinic survey [20], possibly related to statistical power, and plausibly influenced by SHCs’ accessibility at the time. Nevertheless, our findings are similar to this earlier survey in that we found few ethnic differences in sexual healthcare-seeking behaviours, despite observing differences in STI diagnosis [20].

### Meaning and implications

Differences in STI diagnosis rates observed between BC and WBI ethnic groups [14] are unlikely to be explained by the few differences identified in sexual healthcare-seeking and use, according to our study of SHC attendees. In the context of persistent elevated STI risk among England's (and Britain's) BC population [3, 14], we need to ensure their access to sexual healthcare is, at the very least, maintained, if not improved. In terms of STI control, it is encouraging that we found that symptomatic BC women attendees were less likely than WBI women to seek care elsewhere before attending a SHC, because using other services may lengthen care-seeking [17, 27]. It is also encouraging that among those with previous bacterial STI/TV, a higher proportion of BC men than WBI men reported notifying all partners. Yet there is scope to improve such behaviours among all ethnicities, through clinic-based and broader health promotion and structural interventions. It is concerning that BC and WBI women attendees were equally likely to *perceive* themselves at risk of STI, given BC women attendees’ greater likelihood of diagnosis. In a separate analysis of our dataset, heterosexual BC women reported lower recent partner numbers than their WBI counterparts [9], and this lower individual risk behaviour may affect risk perception. However, the picture is complex, with survey data from the general population suggesting little relationship between STI risk perception and both reported unsafe sexual behaviour, and engagement with sexual healthcare [34]. Greater engagement with asymptomatic check-ups is needed, among sexually-active people who are at elevated risk of STI who do not test for STIs, in line with Public Health England’s advice [14].

Since data collection, funding cuts [35] and SHC closures have taken place in England, including some clinics which participated in this study. STI testing across London became accessible online, with an expectation that a significant proportion of patients could test using home self-sampling packs [36]. The effect of these changes on the 4.2% of Londoners of BC ethnicity [10] is unknown; generally, evidence about e-health use by ethnicity is lacking [37]. Evaluation of sexual health service reconfigurations must address impacts on access by ethnicity, to ensure that service changes lessen, or at the very least do not exacerbate, existing health inequalities.

## Conclusions

Although BC people in England are at elevated risk of STI, this elevated risk is unlikely to be explained by differences in sexual healthcare-seeking and use, among those accessing clinics. We found that these behaviours were similar between BC attendees and the WBI ethnic majority. However, differences in reasons for attendance require further exploration, including by using qualitative research methods to unpack the quantitative differences our study has identified, as do the sexual healthcare behaviours of people who do not access SHCs.

## Additional files

Additional file 1: Version of **Table 1.**, showing data for a wider range of ethnic groups (DOCX 28 kb)

Additional file 2: Data for **Figure 1.** (DOCX 17 kb)

Additional file 3: Version of **Figure 1.**’s data, showing data for a wider range of ethnic groups (DOCX 17 kb)

Additional file 4: Version of **Table 2.**, showing data for a wider range of ethnic groups (DOCX 17 kb)

Additional file 5: Version of **Table 3.**, showing data for a wider range of ethnic groups (DOCX 34 kb)

Additional file 6: Text of the online survey (PDF 740 kb)

## Data Availability

The text of our online survey is provided in Additional file 6. The data that support the findings of this study are available from University College London (UCL) but restrictions apply to the availability of these data, which were used under license for the current study, and so are not publicly available. Data are however available from the authors upon reasonable request and with permission of Public Health England.

## Abbreviations

95% CI: 95% confidence interval
AOR: Adjusted odd ratio
BBEST: The Bio-Behavioural Enhanced Surveillance Tool (which our research team developed, and which was used to collect data for the current study)
BC: Black Caribbean
NHS: National Health Service
OR: Odds ratio
PN: Partner notification
SHC: Sexual health clinic
STI: Sexually transmitted infection
TV: *Trichomonas vaginalis*
UK: United Kingdom
WBI: White British or Irish

## Footnotes

1 Throughout this paper we use the terms partner/partnership to refer to sexual partners/partnerships, regardless of their duration, or romantic or social significance.

2 The survey question on reasons for clinic attendance did not include a response option for recent diagnosis or attendance for treatment.

3 See footnote b to Table 1.
