# Supplementary material for: Pathways to, and use of, sexual healthcare among Black Caribbean sexual health clinic attendees in England: evidence from cross-sectional bio-behavioural surveys
Source: BMC Health Serv Res. 2019 Sep 18;19:668. doi: 10.1186/s12913-019-4396-3 (PMC6749649; doi:10.1186/s12913-019-4396-3)
Supplement: Supplementary file 1 — Version of Table 1., showing data for a wider range of ethnic groups (DOCX 28 kb) [file 12913_2019_4396_MOESM1_ESM.docx]

## Version of Table 1, showing data for a wider range of ethnic groups

|  | White British/Irish | White  Other | Black African | Black Caribbean | Indian/  Pakistani/  Bangladeshi | Chinese/  Arab/  Other | Mixed ethnicity | All | p-value |
| --- | --- | --- | --- | --- | --- | --- | --- | --- | --- |
|  | % (95% CI) | % (95% CI) | % (95% CI) | % (95% CI) | % (95% CI) | % (95% CI) | % (95% CI) | % (95% CI) |  |
| **Women**  *Denominator:* | *838* | *335* | *255* | *420* | *157* | *140* | *282* | *2427* |  |
| Age (years) |  |  |  |  |  |  |  |  | 0.019 |
| 15-19 | 9.9% | 6.0% | 14.1% | 12.6% | 14.0% | 9.3% | 19.9% | 11.7% |  |
|  | (5.3-17.7) | (3.8-9.2) | (8.3-23.) | (9.4-16.7) | (9.8-19.7) | (6.7-12.8) | (16.5-23.7) | (8.4-15.9) |  |
| 20-24 | 36.5% | 27.2% | 35.3% | 32.6% | 38.9% | 27.1% | 33.0% | 33.6% |  |
|  | (27.0-47.2) | (20.2-35.5) | (29.8-41.3) | (29.0-36.4) | (28.9-49.9) | (20.3-35.2) | (27.3-39.2) | (27.3-40.5) |  |
| 25-34 | 41.3% | 49.3% | 37.6% | 37.4% | 35.0% | 50.7% | 40.1% | 41.3% |  |
|  | (29.9-53.7) | (41.4-57.1) | (29.3-46.8) | (32.9-42.0) | (26.7-44.4) | (45.8-55.6) | (34.6-45.8) | (34.1-48.9) |  |
| 35+ | 12.3% | 17.6% | 12.9% | 17.4% | 12.1% | 12.9% | 7.1% | 13.4% |  |
|  | (8.4-17.7) | (13.3-23.0) | (8.3-19.6) | (13.5-22.0) | (5.8-23.4) | (8.2-19.7) | (4.3-11.4) | (10.4-17.1) |  |
| Median (IQR) | 25 (21-30) | 27 (23-32) | 25 (21-29) | 26 (22-31) | 24 (21-30) | 26 (22-31) | 24 (20-28) | 25 (21-30) |  |
| Born outside the UK | 8.8% | 92.2% | 59.3% | 23.0% | 23.2% | 63.6% | 21.7% | 33.7% | <0.001 |
|  | (5.3-14.3) | (86-95.8) | (42.1-74.5) | (17.5-29.5) | (16.7-31.3) | (56.9-69.8) | (14.9-30.4) | (29.8-37.7) |  |
| Education (above GCSEs or equivalent) | 84.9% | 84.5% | 82.1% | 77.6% | 89.1% | 83.9% | 76.7% | 82.6% | 0.001 |
|  | (82.1-87.3) | (79.3-88.6) | (75.9-87) | (74.9-80.1) | (85.8-91.7) | (78.6-88.2) | (71-81.6) | (80.3-84.6) |  |
| Any form of employment (yes) | 71.8% | 79.2% | 63.0% | 70.9% | 64.1% | 55.0% | 67.3% | 71.8% | 0.006 |
|  | (60.1-81.1) | (74.4-83.2) | (50.4-74.1) | (66.8-74.7) | (45-79.6) | (48.6-61.2) | (61.1-72.9) | (61.2-77.0) |  |
| Sexual orientation |  |  |  |  |  |  |  |  | 0.138 |
| Straight/heterosexual | 92.4% | 90.1% | 91.8% | 94.3% | 94.9% | 94.3% | 89.4% | 92.2% |  |
|  | (89.3-94.6) | (84.2-94.0) | (87.8-94.5) | (92.8-95.4) | (92.1-96.8) | (88.5-97.3) | (86.7-91.5) | (90.5-93.7) |  |
| Bisexual | 6.3% | 9.3% | 5.5% | 5.0% | 4.5% | 4.3% | 8.9% | 6.5% |  |
|  | (4.1-9.6) | (5.8-14.5) | (3.6-8.4) | (3.7-6.8) | (2.9-6.9) | (1.5-11.9) | (6.7-11.6) | (4.9-8.6) |  |
| Gay/homosexual/lesbian | 0.7% | 0.3% | 0.4% | 0.2% | 0.0% | 0.7% | 0.7% | 0.5% |  |
|  | (0.4-1.4) | (0.0-2.2) | (0.0-3.5) | (0.0-1.4) | (-) | (0.1-7.1) | (0.2-2.7) | (0.4-0.7) |  |
| Rather not say | 0.6% | 0.3% | 2.4% | 0.5% | 0.6% | 0.7% | 1.1% | 0.8% |  |
|  | (0.3-1.2) | (0.0-2.5) | (1.2-4.7) | (0.1-1.5) | (0.2-2.2) | (0.1-3.6) | (0.5-2.4) | (0.5-1.3) |  |
| Number of sexual partners, last 12 months^a^ |  |  |  |  |  |  |  |  | 0.003 |
| 1 | 41.1% | 40.2% | 52.8% | 48.6% | 51.9% | 46.4% | 42.6% | 44.7% |  |
|  | (37.0-45.3) | (35.6-45.0) | (46.1-59.4) | (43.3-53.9) | (38.6-65.0) | (38.0-55.1) | (38.0-47.3) | (40.7-48.6) |  |
| 2 | 18.6% | 19.0% | 25.0% | 23.4% | 24.0% | 20.7% | 23.5% | 21.2% |  |
|  | (14.9-22.8) | (15.6-23.0) | (20.7-29.8) | (18.9-28.6) | (19.9-28.7) | (14.6-28.5) | (18.2-29.7) | (18.6-24.0) |  |
| 3-4 | 23.0% | 20.5% | 16.1% | 19.6% | 14.3% | 16.4% | 18.8% | 19.9% |  |
|  | (18.1-28.8) | (15.6-26.6) | (10.9-23.3) | (14.6-25.8) | (6.7-27.9) | (9.3-27.3) | (13.8-25.1) | (15.8-24.9) |  |
| 5 or more | 17.3% | 20.2% | 6.0% | 8.5% | 9.7% | 16.4% | 15.2% | 14.2% |  |
|  | (13.9-21.4) | (15.8-25.6) | (3.2-11.1) | (6.3-11.2) | (6.7-14.0) | (9.8-26.2) | (10.4-21.5) | (11.9-17.0) |  |
| Any new sexual partners, past 12 months^a^ | 68.6% | 67.7% | 63.2% | 52.9% | 61.4% | 65.2% | 66.3% | 64.3% | 0.002 |
|  | (64.0-72.8) | (60.4-74.1) | (55.3-70.4) | (44.9-60.8) | (52.7-69.5) | (58.7-71.2) | (59.9-72.1) | (60.1-68.2) |  |
| Current partnership(s)^b^ |  |  |  |  |  |  |  |  |  |
| Steady | 63.7%  (57.3-69.7) | 62.6%  (55.5-69.2) | 69.1%  (65.2-72.7) | 62.6%  (59.0-66.0) | 75.4%  (70.0-80.0) | 69.4%  (54.9-80.9) | 68.6%  (58.9-77.0) | 65.6%  (61.8-69.2) | 0.170 |
| Uncommitted but regular | 23.3%  (20.5-26.3) | 21.7%  (16.9-27.3) | 19.8%  (16.1-24.1) | 22.3%  (18.8-26.2) | 11.9%  (6.5-20.8) | 14.9%  (9.3-22.9) | 20.3%  (14.2-28.2) | 20.9%  (18.2-23.9) | 0.070 |
| Casual | 21.4%  (17.4-26.1) | 24.1%  (21.9-26.5) | 16.9%  (12.5-22.5) | 21.7%  (17.6-26.5) | 17.2%  (11.8-24.2) | 20.7%  (14.8-28.0) | 19.5%  (15.3-24.5) | 20.8%  (18.5-23.4) | 0.301 |
| none | 18.7%  (16.2-21.5) | 14.1%  (10.2-19.2) | 17.9%  (13.1-23.9) | 15.7%  (11.3-21.2) | 13.5%  (8.7-20.4) | 13.6%  (10.3-17.7) | 14.8%  (10.8-20.0) | 16.4%  (14.2-18.8) | 0.227 |
| Condom use at last sex with most recent sexual partner^c^ | 28.7%  (25.2-32.5) | 30.0%  (25.3-35.1) | 28.4%  (25.2-32.0) | 31.9%  (24.6-40.2) | 37.8%  (30.5-45.7) | 34.6%  (28.4-41.4) | 27.3%  (19.0-37.5) | 30.2%  (27.2-33.3) | 0.298 |
| Self-perceived risk of STI |  |  |  |  |  |  |  |  | 0.055 |
| Considers self at risk of one or more STIs | 46.1% | 43.1% | 45.3% | 41.7% | 33.3% | 33.1% | 47.3% | 43.4% |  |
|  | (38.9-53.4) | (36.6-49.9) | (36-54.9) | (34.9-48.7) | (23.7-44.5) | (25.4-41.7) | (41.2-53.5) | (37.9-49.1) |  |
| ‘I don’t think I am at risk of getting any STI’^d^ | 53.9% | 56.9% | 54.7% | 58.3% | 66.7% | 66.9% | 52.7% | 56.6% |  |
|  | (46.6-61.1) | (50.1-63.4) | (45.1-64.0) | (51.3-65.1) | (55.5-76.3) | (58.3-74.6) | (46.5-58.8) | (50.9-62.1) |  |
| **Men**  *Denominator* | *573* | *225* | *163* | *207* | *103* | *104* | *131* | *1506* |  |
| Age (years) |  |  |  |  |  |  |  |  | 0.039 |
| 15-19 | 4.7% | 2.7% | 6.7% | 10.1% | 4.9% | 2.9% | 6.9% | 5.4% |  |
|  | (2.5-8.6) | (1.5-4.6) | (3.3-13.3) | (6.8-14.8) | (2.2-10.2) | (0.7-11.5) | (3.9-11.8) | (3.8-7.7) |  |
| 20-24 | 22.0% | 18.7% | 27.0% | 25.1% | 19.4% | 29.8% | 32.8% | 23.8% |  |
|  | (17.3-27.6) | (13.5-25.3) | (20.6-34.5) | (17.8-34.2) | (8.8-37.7) | (22.3-38.6) | (20.9-47.5) | (18.9-29.4) |  |
| 25-34 | 51.8% | 51.1% | 36.2% | 43.0% | 57.3% | 36.5% | 47.3% | 47.7% |  |
|  | (44.5-59.1) | (41.7-60.4) | (29.4-43.6) | (37.2-48.9) | (43.1-70.4) | (28.2-45.7) | (35.8-59.1) | (43.1-52.4) |  |
| 35+ | 21.5% | 27.6% | 30.1% | 21.7% | 18.4% | 30.8% | 13.0% | 23.0% |  |
|  | (14.2-31.0) | (18.1-39.6) | (20.2-42.2) | (13.4-33.3) | (10.5-30.5) | (17.7-47.8) | (6.0-26.0) | (16.5-31.3) |  |
| Median (IQR), years | 26 | 28 | 26 | 26 | 26 | 27 | 25 | 26 |  |
|  | (22-31) | (24-33) | (22-32) | (22-32) | (22-31) | (23-33) | (21-30) | (22-32) |  |
| Born outside the UK | 9.5% | 92.9% | 53.4% | 20.6% | 36.3% | 73.1% | 26.7% | 35.9% | <0.001 |
|  | (6-14.6) | (89.1-95.4) | (34.9-71.0) | (14.7-28.1) | (27.2-46.5) | (66.8-78.6) | (17.9-37.9) | (29.6-42.8) |  |
| Education (above GCSEs or equivalent) | 83.8% | 88.3% | 79.8% | 68.8% | 89.2% | 80.4% | 78.6% | 81.7% | 0.003 |
|  | (78.7-87.9) | (83.5-91.9) | (73.9-84.5) | (62.6-74.4) | (79.8-94.5) | (67.1-89.2) | (69.6-85.5) | (78.6-84.4) |  |
| Any form of employment (yes) | 83.1% | 83.5% | 75.3% | 73.9% | 74.3% | 70.6% | 78.5% | 79.2% | 0.053 |
|  | (77.0-87.9) | (77.4-88.2) | (66.0-82.7) | (64.0-81.8) | (57.6-86.0) | (55.0-82.5) | (68.2-86.1) | (72.6-84.6) |  |
| Sexual orientation |  |  |  |  |  |  |  |  | <0.001 |
| Straight/heterosexual | 75.4% | 59.1% | 93.9% | 87.7% | 85.4% | 67.3% | 77.9% | 77.0% |  |
|  | (64.2-84.0) | (46.4-70.7) | (89.8-96.4) | (79.4-93.0) | (69.5-93.8) | (55.1-77.6) | (57.7-90.1) | (65.7-85.4) |  |
| Bisexual | 4.2% | 6.2% | 0.6% | 5.4% | 5.8% | 6.7% | 6.1% | 4.7% |  |
|  | (2.7-6.4) | (4.0-9.5) | (0.1-4.6) | (3.2-8.8) | (2.0-15.8) | (3.1-14.0) | (3.0-12.1) | (3.2-7.0) |  |
| Gay/homosexual/lesbian | 20.1% | 33.8% | 4.3% | 5.9% | 5.8% | 22.1% | 16.0% | 17.3% |  |
|  | (12.2-31.3) | (23.1-46.4) | (1.9-9.5) | (2.7-12.2) | (2.4-13.7) | (10.9-39.8) | (5.8-37.2) | (10.1-28.0) |  |
| Rather not say | 0.3% | 0.9% | 1.2% | 1.0% | 2.9% | 3.8% | 0.0% | 1.0% |  |
|  | (0.1-1.1) | (0.3-2.4) | (0.4-3.8) | (0.3-3.6) | (0.8-10.0) | (1.4-10.4) | - | (0.6-1.6) |  |
| Number of sexual partners, last 12 months^a^ |  |  |  |  |  |  |  |  | 0.025 |
| 1 | 22.4% | 18.8% | 23.8% | 15.7% | 34.7% | 31.6% | 21.1% | 22.4% |  |
|  | (18.2-27.2) | (12.6-27.1) | (17.1-32.0) | (10.9-22.0) | (27.2-43.1) | (22.5-42.5) | (17.3-25.4) | (19.9-25.1) |  |
| 2 | 14.1% | 10.6% | 20.6% | 20.2% | 12.2% | 10.2% | 10.2% | 14.4% |  |
|  | (9.2-21.0) | (6.6-16.6) | (16.7-25.2) | (16.4-24.6) | (6.8-21.2) | (3.8-24.8) | (6.1-16.3) | (11.6-17.8) |  |
| 3-4 | 24.0% | 18.8% | 20.6% | 25.8% | 26.5% | 19.4% | 26.6% | 23.2% |  |
|  | (19.9-28.6) | (14.2-24.5) | (13.2-30.8) | (20.8-31.4) | (17.3-38.5) | (12.9-28.1) | (20.4-33.8) | (19.8-26.9) |  |
| 5 | 39.5% | 51.8% | 35.0% | 38.4% | 26.5% | 38.8% | 42.2% | 40.0% |  |
|  | (34.4-44.9) | (44.8-58.8) | (29.2-41.3) | (32.3-44.8) | (15.4-41.8) | (30.7-47.6) | (32.8-52.1) | (35.2-45.0) |  |
| Any new sexual partners, past 12 months^a^ | 82.7%  (80.0-85.1) | 84.7%  (77.5-89.8) | 77.7%  (68.3-84.9) | 82.6%  (76.0-87.6) | 73.2%  (62.3-81.8) | 78.1%  (69.9-84.6) | 78.7%  (73.1-83.4) | 81.1%  (78.4-83.6) | 0.127 |
| Current partnership(s)^b^ |  |  |  |  |  |  |  |  |  |
| Steady | 48.7%  (43.8-53.6) | 50.6%  (41.3-59.7) | 53.6%  (49.0-58.2) | 47.3%  (39.4-55.3) | 60.5%  (49.4-70.6) | 46.8%  (31.7-62.4) | 48.1%  (34.4-62.2) | 50.0%  (46.3-53.6) | 0.497 |
| Uncommitted but regular | 21.5%  (18.1-25.3) | 23.9%  (15.1-35.6) | 26.1%  (17.7-36.7) | 35.2%  (28.0-43.1) | 21.0%  (15.6-27.6) | 32.5%  (26.0-39.7) | 31.5%  (26.4-37.0) | 25.8%  (22.3-29.6) | 0.024 |
| Casual | 41.5%  (37.8-5.3) | 40.6%  (34.1-47.4) | 39.1%  (36.0-42.4) | 37.6%  (29.3-46.6) | 37.0%  (26.3-49.2) | 37.7%  (26.8-49.9) | 43.5%  (34.0-53.6) | 40.2%  (36.7-43.8) | 0.757 |
| none | 17.0%  (13.1-21.7) | 19.6%  (14.8-25.6) | 13.2%  (9.5-18.1) | 17.1%  (13.6-21.3) | 20.6%  (16.5-25.4) | 24.5%  (17.6-33.0) | 16.9%  (11.2-24.7) | 17.7%  (15.0-20.9) | 0.150 |
| Condom use at last sex with most recent sexual partner^c^ | 34.4%  (31.0-37.9) | 42.4%  (35.5-49.7) | 39.6%  (31.4-48.5) | 35.1%  (28.5-42.4) | 54.1%  (44.3-63.6) | 47.3%  (36.9-57.8) | 36.8%  (30.8-43.4) | 38.6%  (35.2-42.2) | 0.003 |
| Self-perceived risk of STI |  |  |  |  |  |  |  |  | 0.170 |
| Considers self at risk of one or more STIs | 72.1% | 73.9% | 59.5% | 71.0% | 66.7% | 61.3% | 73.1% | 70.0% |  |
|  | (67.8-76.0) | (63.8-82.0) | (47.7-70.4) | (60.1-80.0) | (54.5-77.0) | (47.9-73.2) | (65.3-79.8) | (66.9-72.9) |  |
| ‘I don’t think I am at risk of getting any STI’^d^ | 27.9% | 26.1% | 40.5% | 29.0% | 33.3% | 38.7% | 26.9% | 30.0% |  |
|  | (24.0-32.2) | (18.0-36.2) | (29.6-52.3) | (20.0-39.9) | (23.0-45.5) | (26.8-52.1) | (20.2-34.7) | (27.1-33.1) |  |

^a^Including opposite- and same-sex partners. Those reporting no sexual partners within the last 12 months were ineligible for the survey, reflecting the STI focus of the research programme.

^b^‘Steady’: married, and/or committed but unmarried. ‘Uncommitted but regular’: not in committed relationship but have sex regularly. ‘Casual’: have sex but not regularly and/or one-off sex partner(s). Participants could select more than one partnership type to reflect their current sexual partnership(s).

^c^Response option ‘we only had oral sex’ was treated as missing, as the (pre-defined) response was ambiguous, for the purpose of this analysis (oral sex on a man can be with or without condoms).

^d^Based on question wording: ‘Thinking about your current sexual lifestyle, which of the following STIs do you think you may be at risk of?’ Response options were a list of STIs including HIV, and a ‘no risk’ response option, as provided in the table.
