# Supplementary material for: Pathways to, and use of, sexual healthcare among Black Caribbean sexual health clinic attendees in England: evidence from cross-sectional bio-behavioural surveys
Source: BMC Health Serv Res. 2019 Sep 18;19:668. doi: 10.1186/s12913-019-4396-3 (PMC6749649; doi:10.1186/s12913-019-4396-3)
Supplement: Supplementary file 2 — Data for Figure 1. (DOCX 17 kb) [file 12913_2019_4396_MOESM2_ESM.docx]

## Data for Figure 1

Multiple reasons could apply.

|  | Women | | | | Men | | | | Comparisons by gender (women as referent) | | | |
| --- | --- | --- | --- | --- | --- | --- | --- | --- | --- | --- | --- | --- |
|  | Black Carib-bean % | White British/ Irish % | OR  (95% CI), p | aOR^a^ (95% CI), p | Black Carib-bean % | White British/ Irish % | OR (95%CI), p | aOR^b^ (95%CI), p | Among Black Caribbean | | Among White British/Irish | |
|  |  |  |  |  |  |  |  |  | OR  (95% CI), p | aOR^c^  (95% CI), p | OR  (95% CI), p | aOR^d^  (95% CI), p |
| *Denominator:* | *420* | *838* |  |  | *207* | *573* |  |  |  |  |  |  |
| ^e^Recent STI diagnosis of any STI (between 6 weeks before, and the day before, clinic attendance). Of which: | 5.0% | 2.0% | p=0.022  2.59  (1.18-5.68) | p=0.007  2.98  (1.43-6.23) | 4.1% | 4.0% | p=0.978  1.02  (0.20-5.23) | p=0.802  1.19  (0.28-5.10) | p=0.664  0.82  (0.30-2.22) | p=0.643  0.79  (0.27-2.35) | p=0.005  2.07  (1.30-3.28) | p=0.029  1.67  (1.06-2.63) |
| *Bacterial STI diagnosis* | 4.1% | 1.3% | p=0.019  3.31  (1.26-8.68) | p=0.009  3.54  (1.45-8.64) | 3.5% | 3.8% | p=0.908  0.92  (0.19-4.36) | p=0.968  1.03  (0.27-3.89) | p=0.691  0.85  (0.36-2.02) | p=0.770  0.88  (0.34-2.27) | p=0.013  3.07  (1.32-7.13) | p=0.031  2.22  (1.09-4.52) |
| *Viral STI diagnosis* | 0.6% | 0.9% | p=0.402  0.69  (0.27-1.75) | p=0.833  0.91  (0.34-2.44) | 0.6% | 0.6% | p=0.982  0.97  (0.08-12.60) | p=0.678  1.58  (0.16-15.97) | p=1.000  1.00  (0.06-16.27) | p=0.994  1.01  (0.07-14.11) | p=0.608  0.71  (0.17-2.98) | p=0.962  0.97  (0.21-4.56) |
| I have (had) symptoms/  My symptoms didn’t go away since the last time I came here for treatment | 41.8% | 35.9% | p=0.080  1.29  (0.97-1.71) | p=0.077  1.33  (0.97-1.83) | 40.5% | 47.4% | p=0.147  0.76  (0.51-1.12) | p=0.081  0.75  (0.54-1.04) | p=0.849  0.95  (0.51-1.75) | p=0.432  0.79  (0.42-1.49) | p=0.093  1.61  (0.91-2.85) | p=0.042  1.53  (1.02-2.29) |
| My partner has (had) symptoms/was diagnosed with an STI | 4.2% | 5.7% | p=0.438  0.73  (0.31-1.71) | p=0.553  0.74  (0.26-2.12) | 17.5% | 10.5% | p=0.011  1.81  (1.18-2.79) | p=0.015  1.82  (1.14-2.90) | p=0.003  4.83  (1.95-11.95) | p=0.014  4.36  (1.42-13.34) | p=0.002  1.94  (1.34-2.80) | p=0.003  1.93  (1.31-2.82) |
| Someone from the clinic contacted me and asked me to come to the clinic | 4.5% | 1.5% | p=0.019  3.16  (1.24-8.02) | p=0.011  3.64  (1.41-9.38) | 2.0% | 2.1% | p=0.884  0.94  (0.37-2.39) | p=0.709  1.20  (0.43-3.38) | p=0.093  0.44  (0.16-1.17) | p=0.100  0.25  (0.04-1.37) | p=0.354  1.48  (0.62-3.52) | p=0.977  1.01  (0.42-2.41) |
| I did not have symptoms but wanted a check-up | 30.7% | 30.1% | p=0.766  1.03  (0.83-1.27) | p=0.391  1.10  (0.88-1.38) | 35.5% | 34.5% | p=0.775  1.05  (0.75-1.47) | p=0.789  1.05  (0.74-1.47) | p=0.101  1.24  (0.95-1.62) | p=0.587  1.12  (0.71-1.78) | p=0.306  1.22  (0.81-1.84) | p=0.928  1.02  (0.67-1.55) |
| I wanted a HIV test | 6.2% | 4.0% | p=0.135  1.58  (0.85-2.94) | p=0.249  1.45  (0.75-2.80) | 11.0% | 11.2% | p=0.938  0.98  (0.58-1.65) | p=0.994  1.00  (0.59-1.70) | p=0.062  1.87  (0.97-3.64) | p=0.383  1.42  (0.61-3.32) | p=0.003  3.02  (1.54-5.93) | p=0.114  1.95  (0.84-4.54) |
| My GP/practice nurse told me to come here | 5.7% | 7.8% | p=0.128  0.72  (0.46-1.12) | p=0.139  0.73  (0.47-1.12) | 3.5% | 5.7% | p=0.249  0.60  (0.24-1.49) | p=0.158  0.58  (0.26-1.27) | p=0.236  0.60  (0.25-1.46) | p=0.482  0.72  (0.27-1.94) | p=0.045  0.72  (0.52-0.99) | p=0.144  0.75  (0.51-1.12) |
| ^f^Contraception or reproductive health reason | 16.6% | 22.4% | p=0.029  0.69  (0.49-0.96) | p=0.019  0.64 (0.45-0.92) | 0.5% | 0.4% | p=0.788  1.41 (0.10-20.70) | p=0.688  1.72  (0.10-29.22) | p=0.008  0.03  (0.00-0.32) | p=0.030  0.04  (0.00-0.70) | p<0.001  0.01  (0.00-0.07) |  |
| ^g^Other reason | 5.2% | 6.1% | p=0.546  0.85  (0.48-1.49) | p=0.277  0.74  (0.41-1.32) | 7.5% | 5.2% | p=0.216  1.49  (0.77-2.90) | p=0.687  1.18  (0.51-2.73) | p=0.124  1.48  (0.88-2.48) | p=0.222  1.94  (0.63-5.90) | p=0.527  0.84  (0.48-1.49) | p=0.988  1.01  (0.52-1.93) |

Notes: Data in this table are survey-reported (more than one reason for attendance was permitted, and so column percentages do not sum to 100%). The exception is recent STI diagnosis (past 6 weeks) which was derived from GUMCAD data. Web-appendix 2 provides a detailed data on multiple ethnic groups.

^a,b,c,d^Adjusted Odds Ratio, adjusted for variables which were statistically significant at p<0.05 in Table 1:

^a^For the inter-ethnic comparison among women: the following binary variables: born in UK, education, sexual orientation, >5/5+ partners in past 12 months (other sexual partner number variables were omitted due to likely covariance).

^b^For the inter-ethnic comparison among men: age as a continuous variable, and the following binary variables: born in UK, education, employment, sexual orientation, reporting regular but uncommitted partner(s).

^c^For the gender comparison among BC attendees: age as a continuous variable, and the following binary variables: education, sexual orientation, >5/5+ partners in past 12 months (other sexual partner number variables were omitted due to likely covariance), reporting any steady partner(s), reporting any regular but uncommitted partner(s), reporting any casual partner(s), and self-perceived STI risk.

^d^For the gender comparison among White British/Irish attendees: age as a continuous variable, and the following binary variables: employment, sexual orientation, >5/5+ partners in past 12 months (other sexual partner number variables were omitted due to likely covariance), reporting any steady partner(s), reporting any casual partner(s), reporting condom use at last sex, and self-perceived STI risk.

^e^We assume that STI diagnosis within the past 6 weeks (i.e. between 6 weeks ago and the day before attendance) would be related to patients’ reasons for attending the clinic (e.g. that they were attending for treatment/PN support, or a follow-up consultation in relation to their recent diagnosis).

^f^Recoded from ‘other’ free-text survey responses.

^g^The remaining ‘other’ variables in this category were diverse.
