# Supplementary material for: Pathways to, and use of, sexual healthcare among Black Caribbean sexual health clinic attendees in England: evidence from cross-sectional bio-behavioural surveys
Source: BMC Health Serv Res. 2019 Sep 18;19:668. doi: 10.1186/s12913-019-4396-3 (PMC6749649; doi:10.1186/s12913-019-4396-3)
Supplement: Supplementary file 3 — Version of Figure 1.’s data, showing data for a wider range of ethnic groups (DOCX 17 kb) [file 12913_2019_4396_MOESM3_ESM.docx]

## Version of Figure 1’s data, showing data for a wider range of ethnic groups

|  | White British/  Irish | White  other | Black African | Black Caribbean | Indian/  Pakistani/  Bangladeshi | Chinese/  Arab/Other | Mixed ethnicity | All | p-value |
| --- | --- | --- | --- | --- | --- | --- | --- | --- | --- |
|  | % (95% CI) | % (95% CI) | % (95% CI) | % (95% CI) | % (95% CI) | % (95% CI) | % (95% CI) | % (95% CI) |  |
| **Women** |  |  |  |  |  |  |  |  |  |
| *Denominator* | *838* | *335* | *255* | *420* | *157* | *140* | *282* | *2427* |  |
| ^a^Recent STI diagnosis (between 6 weeks ago and the day before clinic attendance). Of which: | 2.0%  (1.1-3.6) | 4.7%  (3.0-7.3) | 3.0%  (1.4-6.4) | 5.0%  (2.1-11.3) | 2.2%  (0.8-5.9) | 3.4%  (1.5-7.6) | 6.3%  (3.0-12.8) | 3.6%  (2.0-6.2) | 0.015 |
| *Bacterial STI diagnosis* | 1.3%  (0.7-2.5) | 3.6%  (1.8-7.0) | 1.5%  (0.4-5.7) | 4.1%  (1.5-10.9) | 1.5%  (0.5-4.6) | 2.6%  (1.0-6.6) | 5.4%  (2.6-11.1) | 2.7%  (1.4-5.1) | 0.013 |
| *Viral STI diagnosis* | 0.9%  (0.3-2.4) | 1.8%  (0.9-3.5) | 0.5%  (0.1-3.9) | 0.6%  (0.2-2.0) | 0.7%  (0.1-7.0) | 0.9%  (0.1 -7.3) | 2.1%  (0.6-7.3) | 1.0%  (0.5-2.3) | 0.400 |
| I have (or had) symptoms/My symptoms didn’t go away since the last time I came here for treatment | 35.9%  (24.2-49.5) | 47.1%  (31.5-63.3) | 38.7%  (31.1-46.9) | 41.8%  (32.3-52.1) | 40.8%  (29.8-52.7) | 39.1%  (30.4-48.6) | 41.6%  (34.2-49.4) | 39.9%  (30.0-50.8) | 0.148 |
| My partner has (or had) symptoms/My partner was diagnosed with an STI | 5.7%  (4.4-7.4) | 7.6%  (5.0-11.4) | 5.6%  (2.5-12.2) | 4.2%  (1.9-9.0) | 5.1%  (2.9 -8.7) | 8.7%  (5.1-14.6) | 5.8%  (3.0-10.9) | 5.9%  (4.7-7.3) | 0.513 |
| Someone from the clinic contacted me and asked me to come to the clinic | 1.5%  (0.7-2.9) | 1.5%  (0.8-3.0) | 4.4%  (1.9-9.8) | 4.5%  (1.8 -10.4) | 1.9%  (1.0-3.5) | 0.7%  (0.1-6.3) | 3.3%  (1.8-5.9) | 2.5%  (1.4-4.4) | 0.012 |
| I did not have symptoms but wanted a check-up | 30.1%  (25.8-34.7) | 22.5%  (16.0-30.7) | 26.6%  (21.2-32.8) | 30.7%  (27.4-34.2) | 23.6%  (12.3-40.5) | 21.0%  (12.8-32.6) | 29.2%  (23.1-36.1) | 27.7%  (23.7-32.1) | 0.144 |
| I wanted a HIV test | 4.0%  (2.3-7.0) | 10.9%  (7.9-15.0) | 10.9%  (7.2-16.0) | 6.2%  (3.7-10.3) | 6.4%  (3.5-11.3) | 5.1%  (3.0-8.4) | 4.4%  (2.0-9.3) | 6.3%  (4.4-9.1) | <0.001 |
| My GP/practice nurse told me to come here | 7.8%  (5.9-10.2) | 4.3%  (2.1-8.3) | 4.4%  (3.1-6.3) | 5.7%  (4.0-8.1) | 7.0%  (3.4-14.0) | 8.7%  (5.6-13.3) | 6.2%  (3.4-11.0) | 6.4%  (4.8-8.4) | 0.098 |
| ^b^Contraceptive or reproductive health reason | 22.4%  (10.8-40.8) | 21.6%  (11.1-37.7) | 14.5%  (8.6-23.3) | 16.6%  (8.4-30.0) | 24.8%  (9.8-50.2) | 20.3%  (6.9-46.7) | 17.9%  (9.5-31.1) | 20.0%  (10.0-36.1) | 0.084 |
| ^c^Other reason | 6.1%  (5.1-7.2) | 4.9%  (2.4-9.5) | 9.3%  (5.1-16.4) | 5.2%  (3.4-7.9) | 4.5%  (2.3-8.3) | 8.7%  (5.0-14.7) | 5.8%  (3.5-9.6) | 6.1%  (5.5-6.7) | 0.331 |
| **Men** |  |  |  |  |  |  |  |  |  |
| *Denominator:* | *573* | *225* | *163* | *207* | *103* | *104* | *131* | *1506* |  |
| ^a^Recent STI diagnosis (between 6 weeks ago and the day before clinic attendance). Of which: | 4.0%  (2.4-6.6) | 7.6%  (3.5-15.7) | 3.0%  (1.2-7.3) | 4.1%  (0.9-16.5) | 2.4%  (0.6-8.9) | 3.5%  (0.7-15.8) | 6.6%  (3.1-13.6) | 4.5%  (3.1-6.6) | 0.504 |
| *Bacterial STI diagnosis* | 3.8%  (2.2-6.6) | 5.4%  (2.3-12.5) | 2.3%  (0.9-5.6) | 3.5%  (0.9-13.3) | 2.4%  (0.6-8.9) | 3.5%  (0.7-15.8) | 4.7%  (2.1-10.4) | 3.8%  (2.5-5.8) | 0.762 |
| *Viral STI diagnosis* | 0.6%  (0.2-2.1) | 2.2%  (0.6-7.9) | 0.8%  (0.1-4.4) | 0.6%  (0.1-4.6) | 1.2%  (0.1-9.6) | 1.2%  (0.2-5.5) | 2.8%  (1.1-7.2) | 1.1%  (0.7-1.8) | 0.344 |
| I have (or had) symptoms/My symptoms didn’t go away since the last time I came here for treatment | 47.4%  (38.9-56.1) | 49.8%  (36.0-63.6) | 36.5%  (29.1-44.7) | 40.5%  (32.6-49.0) | 48.0%  (37.5-58.7) | 41.4%  (32.1-51.3) | 44.0%  (28.7-60.5) | 45.0%  (37.5-52.8) | 0.269 |
| My partner has (or had) symptoms/My partner was diagnosed with an STI | 10.5%  (8.2-13.4) | 11.8%  (8.8-15.6) | 13.5%  (8.5-20.7) | 17.5%  (11.3-26.2) | 7.0%  (3.0-15.4) | 9.1%  (4.9-16.3) | 16.8%  (11.2-24.4) | 12.2%  (9.7-15.1) | 0.053 |
| Someone from the clinic contacted me and asked me to come to the clinic | 2.1%  (1.1-4.0) | 3.6%  (1.6-8.0) | 1.9%  (0.7-5.0) | 2.0%  (1.1-3.8) | 2.0%  (0.7-5.3) | 3.0%  (1.3-6.7) | 3.2%  (1.5-6.9) | 2.5%  (1.6-3.8) | 0.562 |
| I did not have symptoms but wanted a check-up | 34.5%  (26.1- 43.9) | 31.7%  (22.4-42.7) | 31.4%  (25.9-37.5) | 35.5%  (29.3-42.3) | 37.0%  (25.0-50.9) | 32.3%  (26.0-39.3) | 40.8%  (30.5-52.0) | 34.4%  (28.5-40.9) | 0.586 |
| I wanted a HIV test | 11.2%  (8.6-14.4) | 14.9%  (9.4-22.9) | 18.6%  (13.7-24.7) | 11.0%  (7.9-15.2) | 18.0%  (11.1-27.9) | 13.1%  (7.3-22.5) | 11.2%  (5.8-20.6) | 13.1%  (10.5-16.2) | 0.124 |
| My GP/practice nurse told me to come here | 5.7%  (4.1-7.8) | 6.8%  (3.9-11.5) | 5.8%  (3.3-10.0) | 3.5%  (1.6-7.4) | 6.0%  (1.9-17.7) | 12.1%  (5.9-23.3) | 3.2%  (1.3-7.5) | 5.8%  (4.2-7.9) | 0.124 |
| ^b^Contraceptive or reproductive health reason | 0.4%  (0.1-1.5) | 0.5%  (0.1-3.5) | 0.6%  (0.1-4.9) | 0.5%  (0.1-4.4) | 0.0%  - | 0.0%  - | 0.8%  (0.1-7.5) | 0.4%  (0.2-0.9) | 0.941 |
| ^c^Other reason | 5.2%  (3.1-8.3) | 5.9%  (3.5-9.6) | 8.3%  (4.3-15.4) | 7.5%  (4.7-11.7) | 6.0%  (1.7-19.5) | 9.1%  (5.5-14.7) | 4.0%  (1.4-10.7) | 6.1%  (4.7-8.1) | 0.484 |

Multiple reasons could apply.

^a^We assume that STI diagnosis within the past 6 weeks (i.e. between 6 weeks ago and the day before attendance) would be related to patients’ reasons for attending the clinic (e.g. that they were attending for treatment/PN support, or a follow-up consultation in relation to their recent diagnosis).

^b^Recoded from ‘other’ free-text survey responses.

^c^The remaining ‘other’ variables in this category were diverse.
