# Supplementary material for: Pathways to, and use of, sexual healthcare among Black Caribbean sexual health clinic attendees in England: evidence from cross-sectional bio-behavioural surveys
Source: BMC Health Serv Res. 2019 Sep 18;19:668. doi: 10.1186/s12913-019-4396-3 (PMC6749649; doi:10.1186/s12913-019-4396-3)
Supplement: Supplementary file 4 — Version of Table 2., showing data for a wider range of ethnic groups (DOCX 17 kb) [file 12913_2019_4396_MOESM4_ESM.docx]

## Version of Table 2, showing data for a wider range of ethnic groups

|  | White British/Irish | White  other | Black African | Black Caribbean | Indian/  Pakistani/  Bangladeshi | Chinese/  Arab/Other | Mixed ethnicity | All | p-value |
| --- | --- | --- | --- | --- | --- | --- | --- | --- | --- |
|  | % (95% CI) | % (95% CI) | % (95% CI) | % (95% CI) | % (95% CI) | % (95% CI) | % (95% CI) | % (95% CI) |  |
| **Women** |  |  |  |  |  |  |  |  |  |
| *Denominator* | *296* | *155* | *96* | *169* | *64* | *54* | *114* | *948* |  |
| How long ago did your symptoms start? |  |  |  |  |  |  |  |  | 0.625 |
| In the last 7 days | 32.8%  (28.8-37.0) | 40.9%  (30.5-52.2) | 34.4%  (28.6-40.7) | 36.9%  (29.1-45.5) | 35.9%  (22.1-52.5) | 35.2%  (17.5-58.2) | 34.5%  (26.0-44.2) | 35.6%  (31.8-39.5) |  |
| 1-2 weeks ago | 20.9%  (17.3-25.1) | 18.8%  (11.7-29.0) | 17.7%  (11.9-25.6) | 23.8%  (16.6-32.9) | 18.8%  (12.2-27.7) | 18.5%  (11.1-29.3) | 23.9%  (19.9-28.4) | 20.8%  (18.3-23.7) |  |
| More than 2 weeks ago but in the last month | 16.2%  (13.4-19.5) | 13.0%  (9.5-17.5) | 15.6%  (8.6-26.6) | 13.7%  (9.9-18.7) | 21.9%  (11.2-38.2) | 27.8%  (18.4-39.7) | 15.9%  (12.7-19.8) | 16.2%  (14.4-18.2) |  |
| More than 1 month ago but in the last 3 months | 14.9%  (12.1-18.2) | 14.3%  (6.0-30.5) | 18.8%  (13.2-25.9) | 13.7%  (10.6-17.6) | 7.8%  (1.9-27.1) | 9.3%  (3.9-20.2) | 14.2%  (8.3-23.2) | 14.1%  (10.8-18.1) |  |
| More than 3 months ago but in the last 12 months | 8.8%  (6.1-12.5) | 9.1%  (4.8-16.7) | 10.4%  (5.6-18.5) | 8.3%  (3.8-17.1) | 10.9%  (6.5-17.7) | 5.6%  (1.9-15.0) | 8.8%  (5.0-15.3) | 8.9%  (7.0-11.3) |  |
| More than 12 months ago | 6.4%  (4.2-9.8) | 3.9%  (2.0-7.4) | 3.1%  (0.8-10.9) | 3.6%  (1.2-10.2) | 4.7%  (0.9-20.4) | 3.7%  (0.6-20.1) | 2.7%  (0.9-7.6) | 4.4%  (3.3-5.9) |  |
| Before coming here today, did you try to get treatment or advice for your symptoms from anywhere else? (Yes) | 45.2%  (39.7-50.9) | 47.7%  (42.3-53.2) | 28.1%  (20.3-37.6) | 35.1%  (25.9-45.6) | 46.9%  (28.0-66.6) | 50.9%  (39.4-62.4) | 39.3%  (31.4-47.7) | 41.8%  (37.4-46.3) | 0.043 |
| Since your symptoms started, have you had sex? |  |  |  |  |  |  |  |  | 0.201 |
| No | 47.5%  (40.3-54.7) | 48.7%  (41.0-56.4) | 51.0%  (41.3-60.7) | 57.5%  (51.1-63.6) | 35.9%  (23.3-50.9) | 50.9%  (31.4-70.2) | 50.0%  (39.2-60.8) | 49.5%  (45.5-53.6) |  |
| Yes, with only one partner | 45.4%  (37.3-53.8) | 44.7%  (38.6-51.0) | 49.0%  (39.3-58.7) | 40.1%  (34.8-45.7) | 56.3%  (47.2-64.9) | 45.3%  (24.7-67.7) | 46.4%  (37.7-55.4) | 45.6%  (41.8-49.4) |  |
| Yes, with more than one partner | 7.1%  (4.2-11.9) | 6.6%  (4.3-9.9) | 0.0%  (-) | 2.4%  (0.8-6.7) | 7.8%  (2.2-23.9) | 3.8%  (1.0-13.2) | 3.6%  (1.2-10.3) | 4.9%  (3.4-7.1) |  |
| *Any sex (i.e. one or more partners)* | 52.5%  (45.3-59.7) | 51.3%  (43.6-59.0) | 49.0%  (39.3-58.7) | 42.5%  (36.4-48.9) | 64.1%  (49.1-76.7) | 49.1%  (29.8-68.6) | 50.0%  (39.2-60.8) | 50.5%  (46.4-54.5) | 0.230 |
| **Men** |  |  |  |  |  |  |  |  |  |
| *Denominator* | *267* | *110* | *57* | *81* | *48* | *41* | *55* | *659* |  |
| How long ago did your symptoms start? |  |  |  |  |  |  |  |  | 0.784 |
| In the last 7 days | 34.6%  (29.0-40.6) | 38.2%  (26.9-50.9) | 43.9%  (30.6-58.1) | 44.4%  (27.3-63.0) | 35.4%  (24.4-48.2) | 35.0%  (20.4-53.1) | 36.4%  (22.9-52.4) | 37.4%  (32.4-42.8) |  |
| 1-2 weeks ago | 19.5%  (16.8-22.6) | 20.9%  (13.3-31.4) | 21.1%  (12.3-33.7) | 22.2%  (11.9-37.7) | 18.8%  (8.8-35.7) | 15.0%  (6.1-32.5) | 14.5%  (6.9-28.1) | 19.5%  (17.5-21.7) |  |
| More than 2 weeks ago but in the last month | 16.5%  (12.8-21.2) | 17.3%  (12.7-23.0) | 10.5%  (4.0-25.1) | 7.4%  (1.7-27.6) | 12.5%  (5.5-26.1) | 17.5%  (6.7-38.7) | 10.9%  (5.5-20.6) | 14.3%  (11.2-18.2) |  |
| More than 1 month ago but in the last 3 months | 11.7%  (7.8-17.2) | 10.0%  (4.2-22.1) | 8.8%  (2.4-27.4) | 9.9%  (4.5-20.2) | 12.5%  (8.0-18.9) | 15.0%  (6.7-30.4) | 21.8%  (12.2-36.0) | 12.0%  (8.8-16.2) |  |
| More than 3 months ago but in the last 12 months | 9.8%  (6.2-15.0) | 7.3%  (3.1-16.3) | 8.8%  (3.2-21.8) | 9.9%  (5.5-17.1) | 14.6%  (4.6-37.5) | 12.5%  (3.7-34.6) | 10.9%  (3.5-29.2) | 9.9%  (8.0-12.2) |  |
| More than 12 months ago | 7.9%  (5.2-11.7) | 6.4%  (1.7-21.4) | 7.0%  (3.2-14.6) | 6.2%  (2.3-15.6) | 6.3%  (1.8-19.4) | 5.0%  (1.0-21.7) | 5.5%  (2.0-14.2) | 6.8%  (5.0-9.2) |  |
| Before coming here today, did you try to get treatment or advice for your symptoms from anywhere else? (Yes) | 35.3%  (27.7-43.8) | 32.4%  (19.3-49.1) | 26.3%  (17.9-37.0) | 30.0%  (20.8-41.1) | 27.7%  (14.1-47.2) | 30.0%  (13.8-53.4) | 30.9%  (19.8-44.7) | 32.2%  (25.6-39.5) | 0.760 |
| Since your symptoms started, have you had sex? |  |  |  |  |  |  |  |  | 0.587 |
| No | 62.6%  (51.3-72.8) | 56.9%  (42.2-70.4) | 66.7%  (53.8-77.5) | 61.7%  (49.8-72.4) | 55.3%  [37.7-71.7) | 60.0%  (46.0-72.6) | 54.5%  (39.5-68.8) | 60.6%  (51.6-68.9) |  |
| Yes, with only one partner | 31.7%  (22.7-42.3) | 34.9%  (25.0-46.3) | 21.1%  (14.4-29.7) | 28.4%  (21.0-37.2) | 36.2%  (23.1-51.6) | 32.5%  (21.1-46.5) | 36.4%  (21.5-54.4) | 31.7%  (24.9-39.2) |  |
| Yes, with more than one partner | 5.7%  (3.6-8.9) | 8.3%  (2.3-25.3) | 12.3%  (7.6-19.2) | 9.9%  (3.7-24.0) | 8.5%  (3.0-21.9) | 7.5%  (1.9-24.9) | 9.1%  (3.7-20.7) | 7.8%  (5.1-11.8) |  |
| *Any sex (i.e. one or more partners)* | 37.4%  (27.2-48.7) | 43.1%  (29.6-57.8) | 33.3%  (22.5-46.2) | 38.3%  (27.6-50.2) | 44.7%  (28.3-62.3) | 40.0%  (27.4-54.0) | 45.5%  (31.2-60.5) | 39.4%  (31.1-48.4) | 0.601 |
