# Supplementary material for: Pathways to, and use of, sexual healthcare among Black Caribbean sexual health clinic attendees in England: evidence from cross-sectional bio-behavioural surveys
Source: BMC Health Serv Res. 2019 Sep 18;19:668. doi: 10.1186/s12913-019-4396-3 (PMC6749649; doi:10.1186/s12913-019-4396-3)
Supplement: Supplementary file 5 — Version of Table 3., showing data for a wider range of ethnic groups (DOCX 34 kb) [file 12913_2019_4396_MOESM5_ESM.docx]

## Version of Table 3, showing data for a wider range of ethnic groups

|  | White British/Irish | White  other | Black African | Black Caribbean | Indian/  Pakistani/  Bangladeshi | Chinese/  Arab/Other | Mixed ethnicity | All | p-value |
| --- | --- | --- | --- | --- | --- | --- | --- | --- | --- |
| **Women** | % (95% CI) | % (95% CI) | % (95% CI) | % (95% CI) | % (95% CI) | % (95% CI) | % (95% CI) | % (95% CI) |  |
| **Of all participants:**  *Denominator:* | *838* | *335* | *255* | *420* | *157* | *140* | *282* | *2427* |  |
| Ever before diagnosed with or treated for an STI (self-report)^a^ | 40.0%  (31.7-48.9) | 40.3%  (35.3-45.5) | 47.5%  (37.5-57.6) | 61.2%  (52.4-69.3) | 25.5%  (18.3-34.2) | 42.1%  (34.6-50.1) | 57.1%  (46.7-66.9) | 45.7%  (38.4-53.1) | <0.001 |
| **Of those reporting previous STI diagnosis/treatment^a^**  *Denominator:* | *335* | *135* | *121* | *257* | *40* | *59* | *161* | *1108* | *335* |
| Last time this happened: |  |  |  |  |  |  |  |  | 0.083 |
| In the last 7 days | 7.2%  (4.7-10.7) | 7.4%  (3.9-13.6) | 3.3%  (1.4-7.6) | 5.4%  (2.0-14.1) | 5.0%  (2.1-11.4) | 1.7%  (0.2-15.2) | 3.7%  (1.4-9.5) | 5.5%  (3.5-8.6) |  |
| 1-2 weeks ago | 1.5%  (0.7-3.2) | 3.0%  (1.3-6.5) | 4.1%  (2.2-7.8) | 1.2%  (0.2-5.5) | 5.0%  (1.8-12.9) | 10.2%  (3.7-25.2) | 2.5%  (0.5-10.5) | 2.6%  (1.7-3.9) |  |
| More than 2 weeks ago but in the last month | 2.1%  (1.2-3.5) | 3.7%  (1.9-7.1) | 6.6%  (4.0-10.8) | 1.6%  (0.7-3.5) | 2.5%  (0.2-21.3) | 10.2%  (5.1-19.3) | 6.8%  (3.1-14.2) | 3.8%  (2.9-4.9) |  |
| More than 1 month ago but in the last 3 months | 9.3%  (6.3-13.4) | 11.9%  (7.9-17.3) | 9.1%  (5.4-14.9) | 7.8%  (4.9-12.0) | 10.0%  (5.4-17.8) | 13.6%  (7.8-22.6) | 8.1%  (4.9-13.1) | 9.3%  (7.8-11.0) |  |
| More than 3 months ago but in the last 12 months | 24.5%  (18.0-32.3) | 25.2%  (16.5-36.4) | 19.8%  (13.6-27.9) | 22.6%  (18.0-27.9) | 25.0%  (13.1-42.5) | 25.4%  (16.9-36.4) | 21.7%  (15.7-29.3) | 23.3%  (20.3-26.5) |  |
| More than 12 months ago | 55.5%  (43.1-67.3) | 48.9%  (39.5-58.3) | 57.0%  (49.0-64.7) | 61.5%  (52.7-69.6) | 52.5%  (41.1-63.6) | 39.0%  (26.7-52.9) | 57.1%  (45.7-67.9) | 55.5%  (49.7-61.2) |  |
| STI(s) diagnosed/treated at last episode^b^ |  |  |  |  |  |  |  |  | 0.358 |
| Chlamydia | 54.9%  (47.9-61.8) | 44.4%  (27.7-62.6) | 56.2%  (43.0-68.6) | 57.6%  (48.2-66.5) | 42.5%  (26.5-60.2) | 50.8%  (34.4-67.1) | 54.0%  (46.9-61.0) | 53.6%  (46.9-60.2) |  |
| Gonorrhoea | 9.3%  (6.5-13.0) | 3.7%  (1.4-9.4) | 12.4%  (8.2-18.4) | 11.3%  (8.6-14.6) | 12.5%  (5.7-25.1) | 11.9%  (4.3-28.9) | 14.3%  (10.4-19.4) | 10.4%  (8.4-12.7) | 0.104 |
| Genital warts | 19.4%  (14.1-26.1) | 8.9%  (4.9-15.6) | 9.9%  (6.4-15.0) | 5.1%  (2.5-10.1) | 10.0%  (4.0-23.0) | 11.9%  (5.7-23.0) | 11.2%  (8.2-15.1) | 11.8%  (8.8-15.6) | <0.001 |
| Syphilis | 1.2%  (0.7-2.1) | 1.5%  (0.3-6.1) | 3.3%  (1.5-6.9) | 1.6%  (0.8-3.0) | 1.2%  (0.7-2.1) | 1.5%  (0.3-6.1) | 3.3%  (1.5-6.9) | 1.6%  (0.8-3.0) | 0.094 |
| *Trichomonas vaginalis* (Trich, TV) | 3.6%  (1.9-6.6) | 5.9%  (2.9-11.8) | 11.6%  (5.7-22.1) | 12.8%  (10.8-15.2) | 10.0%  (6.1-16.0) | 6.8%  (3.5-12.8) | 9.9%  (7.9-12.5) | 8.2%  (6.8-9.9) | 0.007 |
| Genital herpes | 14.0%  (10.3-18.9) | 17.8%  (13.3-23.3) | 11.6%  (6.8-18.9) | 10.9%  (8.2-14.4) | 10.0%  (2.7-31.1) | 15.3%  (8.9-25.0) | 10.6%  (7.4-14.8) | 12.9%  (11.4-14.6) | 0.357 |
| Hepatitis B | 0.9%  (0.3-2.3) | 0.7%  (0.1-5.2) | 2.5%  (1.0-6.2) | 0.4%  (0.1-2.5) | 5.0%  (2.1-11.4) | 5.1%  (1.7-14.6) | 2.5%  (1.3-4.6) | 1.5%  (0.9-2.5) | 0.024 |
| NSU/NGU (non-specific urethritis/non-gonococcal urethritis) | 0.9%  (0.3-2.3) | 0.7%  (0.1-5.7) | 2.5%  (0.7-8.5) | 1.2%  (0.3-4.3) | 7.5%  (2.3-21.7) | 1.7%  (0.3-9.0) | 2.5%  (0.9-6.7) | 1.6%  (1.0-2.6) | 0.101 |
| Epididymitis | 0.3%  (0.0-2.9) | 0.0%  (-) | 1.7%  (0.5-4.9) | 0.4%  (0.1-2.5) | 0.0%  (-) | 1.7%  (0.3-9.0) | 1.2%  (0.4-3.6) | 0.6%  (0.2-1.6) | 0.435 |
| HIV | 1.8%  (1.1-3.0) | 1.5%  (0.4-5.3) | 2.5%  (1.2-4.9) | 1.2%  (0.5-2.7) | 5.0%  (1.0-21.5) | 6.8%  (2.9-14.9) | 3.1%  (1.3-7.5) | 2.3%  (1.6-3.1) | 0.077 |
| Cannot remember which STI | 7.8%  (5.8-10.4) | 19.3%  (11.5-30.4) | 10.7%  (5.6-19.5) | 12.1%  (8.9-16.1) | 20.0%  (8.6-39.8) | 22.0%  (10.2-41.3) | 14.3%  (9.6-20.7) | 12.6%  (10.8-14.7) | 0.053 |
| **Of those reporting diagnosis/treatment of bacterial STI(s) or trichomoniasis, at last STI episode^c^**  *Denominator:* |  |  |  |  |  |  |  |  |  |
| At that time, did the clinic staff advise you to inform your sexual partners to test for STIs/come to clinic? |  |  |  |  |  |  |  |  | 0.460 |
| No | 9.5%  (4.8-17.8) | 5.9%  (2.0-15.9) | 9.5%  (4.1-20.6) | 8.7%  (6.3-11.9) | 20.0%  (8.7-39.7) | 12.1%  (5.3-25.5) | 5.5%  (3.0-9.8) | 8.8%  (6.9-11.2) |  |
| Yes | 83.6%  (72.2-90.9) | 86.8%  (77.5-92.6) | 83.3%  (75.4-89.1) | 84.2%  (78.8-88.5) | 80.0%  (60.3-91.3) | 84.8%  (73.0-92.1) | 84.4%  (79.3-88.4) | 84.1%  (81.9-86.1) |  |
| Can’t remember | 7.0%  (3.1-15.0) | 7.4%  (3.2-15.8) | 7.1%  (3.8-13.1) | 7.1%  (4.3-11.4) | 0.0%  (-) | 3.0%  (0.6-13.1) | 10.1%  (6.6-15.1) | 7.1%  (5.4-9.2) |  |
| At that time, did you inform your sexual partners to test/take treatment for STIs? |  |  |  |  |  |  |  |  | 0.139 |
| No, I didn't tell any partners | 10.9%  (7.8-15.1) | 4.4%  (1.8-10.4) | 7.1%  (2.3-20.0) | 6.0%  (4.3-8.3) | 12.0%  (5.0-26.0) | 15.2%  (5.6-34.7) | 8.3%  (4.7-14.1) | 8.4%  (6.7-10.5) |  |
| Yes, I told ALL my partners | 73.6%  (67.7-78.8) | 79.4%  (66.5-88.2) | 85.7%  (75.9-91.9) | 80.4%  (71.3-87.2) | 68.0%  (42.8-85.8) | 66.7%  (44.0-83.6) | 78.9%  (70.2-85.6) | 77.7%  (74.1-80.9) |  |
| Can't remember | 8.5%  (5.0-14.0) | 8.8%  (4.4-16.8) | 2.4%  (0.7-8.1) | 7.1%  (3.5-13.8) | 0.0%  (-) | 0.0%  (-) | 7.3%  (4.5-11.7) | 6.5%  (4.9-8.7) |  |
| Yes, I told SOME of my partners | 7.0%  (5.2-9.4) | 7.4%  (3.2-15.8) | 4.8%  (1.6-13.7) | 6.5%  (2.9-14.1) | 20.0%  (6.7-46.5) | 18.2%  (4.7-49.8) | 5.5%  (2.8-10.5) | 7.4%  (4.9-11.0) |  |
| **Of those diagnosed with/treated bacterial STI/Trich,**  **AND who informed some/all partners to test/come to the clinic^c^**  *Denominator:* | *162* | *59* | *76* | *160* | *22* | *28* | *92* | *599* |  |
| At that time, how did you inform you sexual partners to test for STIs/come to clinic?^b^ |  |  |  |  |  |  |  |  |  |
| In person | 56.8%  (44.6-68.2) | 74.6%  (68.0-80.2) | 59.2%  (45.5-71.6) | 66.9%  (60.6-72.6) | 50.0%  (34.6-65.4) | 67.9%  (48.8-82.4) | 69.6%  (63.9-74.7) | 63.8%  (59.3-68.0) | 0.085 |
| Via a text message | 29.0%  (22.6-36.4) | 25.4%  (16.7-36.6) | 17.1%  (11.6-24.5) | 19.4%  (14.9-24.8) | 27.3%  (18.7-38.0) | 25.0%  (11.7-45.6) | 21.7%  (15.9-29.1) | 23.2%  (20.7-25.9) | 0.188 |
| Via an email | 1.2%  (0.2-9.0) | 0.0%  (-) | 0.0%  (-) | 0.0%  (-) | 0.0%  (-) | 0.0%  (-) | 0.0%  (-) | 0.3%  (0.0-2.4) | 0.644 |
| Via telephone | 26.5%  (20.5-33.6) | 20.3%  (11.8-32.8) | 42.1%  (30.8-54.3) | 32.5%  (28.1-37.3) | 27.3%  (13.0-48.5) | 14.3%  (3.8-41.3) | 27.2%  (20.3-35.3) | 29.0%  (24.0-34.7) | 0.031 |
| Via social media | 0.6%  (0.1-5.7) | 1.7%  (0.2-11.1) | 0.0%  (-) | 1.3%  (0.1-10.0) | 0.0%  (-) | 0.0%  (-) | 1.1%  (0.1-7.5) | 0.8%  (0.3-2.6) | 0.890 |
| Via a friend | 0.0%  (-) | 0.0%  (-) | 1.3%  (0.2-9.6) | 0.0%  (-) | 0.0%  (-) | 0.0%  (-) | 1.1%  (0.1-8.1) | 0.3%  (0.1-1.3) | 0.570 |
| Via a clinic health adviser/clinic staff | 2.5%  (0.7-8.8) | 0.0%  (-) | 0.0%  (-) | 3.1%  (1.7-5.5) | 4.5%  (0.8-21.5) | 0.0%  (-) | 4.3%  (0.9-18.1) | 2.3%  (1.4-3.8) | 0.502 |
| Other | 1.2%  (0.5-3.2) | 0.0%  (-) | 0.0%  (-) | 0.6%  (0.1-3.7) | 0.0%  (-) | 3.6%  (0.4-25.5) | 4.3%  (1.9-9.8) | 1.3%  (0.8-2.3) | 0.159 |
| **Of those diagnosed with/treated bacterial STI/trichomoniasis,**  **AND who did NOT inform ALL of their partners to test/attend clinic (informed none, or informed some)^b,c^** |  |  |  |  |  |  |  |  |  |
| *Denominator:* | *36* | *8* | *10* | *23* | *8* | *11* | *15* | *111* |  |
| At that time, why did you not inform (some of) your sexual partners to test for the infection/come to the clinic?^d^ | N | n | n | n | n | n | n | n |  |
| My partner(s) lives outside the UK | 0 | 0 | 0 | 1 | 0 | 2 | 0 | 3 |  |
| I was embarrassed to tell my partner(s) about the infection | 8 | 3 | 1 | 7 | 2 | 2 | 4 | 27 |  |
| I was scared of telling my partner(s) about the infection | 3 | 0 | 1 | 7 | 0 | 1 | 3 | 15 |  |
| I was worried that my partner(s) would leave me | 1 | 0 | 0 | 2 | 0 | 1 | 0 | 4 |  |
| I did not have contact details of my partner(s) | 12 | 2 | 1 | 6 | 2 | 0 | 3 | 26 |  |
| I was not too concerned about telling my main partner | 2 | 0 | 0 | 0 | 1 | 1 | 2 | 6 |  |
| I was not too concerned about telling my casual / one-off partner(s) | 11 | 0 | 0 | 6 | 2 | 3 | 3 | 25 |  |
| Other | 9 | 4 | 6 | 5 | 2 | 5 | 4 | 35 |  |
| **Men** | % (95% CI) | % (95% CI) | % (95% CI) | % (95% CI) | % (95% CI) | % (95% CI) | % (95% CI) | % (95% CI) |  |
| **Of all participants**  *Denominator:* | *573* | *225* | *163* | *207* | *103* | *104* | *131* | *1506* |  |
| Ever before diagnosed with an STI (self-report)^a^ | 50.6%  (43.3-57.9) | 49.8%  (40.3-59.3) | 55.2%  (44.7-65.3) | 60.9%  (50.6-70.3) | 38.8%  (25.4-54.3) | 41.3%  (30.0-53.7) | 55.7%  (46.5-64.6) | 51.4%  (44.3-58.4) | 0.041 |
| **Of those reporting previous STI diagnosis/treatment^a^**  *Denominator:* | *290* | *112* | *90* | *126* | *40* | *43* | *73* | *774* |  |
| Last time this happened |  |  |  |  |  |  |  |  | 0.654 |
| In the last 7 days | 6.2%  (3.4-10.9) | 5.4%  (2.4-11.7) | 3.3%  (1.0-10.9) | 4.0%  (1.0-14.2) | 0.0%  (-) | 4.7%  (1.4-14.0) | 8.2%  (4.8-13.8) | 5.2%  (3.0-8.7) |  |
| 1-2 weeks ago | 4.5%  (2.7-7.4) | 7.1%  (4.1-12.1) | 3.3%  (1.0-10.6) | 5.6%  (2.3-12.8) | 7.5%  (1.7-27.2) | 2.3%  (0.5-10.8) | 4.1%  (1.5-10.8) | 4.9%  (3.8-6.4) |  |
| More than 2 weeks ago but in the last month | 5.2%  (3.3-8.0) | 7.1%  (3.9-12.7) | 4.4%  (1.7-11.3) | 5.6%  (2.3-12.8) | 5.0%  (1.3-17.8) | 4.7%  (1.4-14.7) | 4.1%  (1.2-13.0) | 5.3%  (3.7-7.6) |  |
| More than 1 month ago but in the last 3 months | 9.0%  (5.0-15.5) | 9.8%  (5.9-16.0) | 6.7%  (2.9-14.4) | 7.9%  (3.4-17.4) | 10.0%  (3.8-24.0) | 14.0%  (6.0-29.3) | 11.0%  (5.8-19.6) | 9.2%  (7.4-11.3) |  |
| More than 3 months ago but in the last 12 months | 21.7%  (16.1-28.6) | 28.6%  (17.3-43.3) | 20.0%  (13.3-28.9) | 27.8%  (23.3-32.7) | 32.5%  (22.5-44.4) | 20.9%  (11.5-35.1) | 28.8%  (19.1-40.8) | 24.7%  (20.8-29.0) |  |
| More than 12 months ago | 53.4%  (47.6-59.2) | 42.0%  (28.8-56.3) | 62.2%  (52.3-71.2) | 49.2%  (38.9-59.6) | 45.0%  (33.1-57.5) | 53.5%  (41.7-64.9) | 43.8%  (32.6-55.8) | 50.8%  (46.1-55.5) |  |
| STI(s) diagnosed/treated at last episode^b^ |  |  |  |  |  |  |  |  |  |
| Chlamydia | 31.7%  (28.9-34.7) | 30.4%  (25.3-35.9) | 54.4%  (43.2-65.2) | 55.6%  (45.6-65.0) | 30.0%  (15.7-49.6) | 44.2%  (32.1-57.0) | 46.6%  (30.7-63.2) | 40.1%  (35.0-45.4) | 0.001 |
| Gonorrhoea | 17.2%  (13.9-21.2) | 26.8%  (17.8-38.1) | 8.9%  (6.7-11.8) | 19.8%  (15.0-25.8) | 12.5%  (5.7-25.3) | 14.0%  (7.6-24.2) | 32.9%  (21.6-46.5) | 19.1%  (15.2-23.7) | 0.001 |
| Genital warts | 21.4%  (16.2-27.7) | 8.0%  (4.8-13.1) | 5.6%  (3.1-9.8) | 7.1%  (3.6-13.6) | 12.5%  (7.3-20.7) | 16.3%  (5.0-42.0) | 13.7%  (7.3-24.3) | 13.8%  (10.9-17.3) | 0.003 |
| Syphilis | 5.9%  (3.5-9.6) | 11.6%  (6.0-21.3) | 5.6%  (2.0-14.6) | 4.0%  (1.4-10.6) | 7.5%  (3.7-14.7) | 11.6%  (6.3-20.6) | 5.5%  (2.0-14.3) | 6.7%  (4.6-9.7) | 0.190 |
| *Trichomonas vaginalis* (Trich, TV) | 0.0%  (-) | 0.0%  (-) | 0.0%  (-) | 0.8%  (0.1-5.9) | 0.0%  (-) | 2.3%  (0.2-20.0) | 1.4%  (0.1-12.5) | 0.4%  (0.1-1.9) | 0.651 |
| Genital herpes | 7.6%  (4.7-12.1) | 8.9%  (4.9-15.6) | 3.3%  (1.2-8.8) | 3.2%  (0.8-11.2) | 7.5%  (2.2-22.5) | 7.0%  (2.4-18.7) | 9.6%  (4.8-18.1) | 6.7%  (5.4-8.3) | 0.388 |
| Hepatitis B | 1.0%  (0.4-2.9) | 0.0%  (-) | 0.0%  (-) | 1.6%  (0.4-6.3) | 2.5%  (0.4-13.3) | 2.3%  (0.2-20.0) | 1.4%  (0.2-9.1) | 1.0%  (0.6-1.8) | 0.703 |
| NSU/NGU (non-specific urethritis/non-gonococcal urethritis) | 7.9%  (5.0-12.3) | 7.1%  (5.4-9.4) | 7.8%  (3.9-15.0) | 15.1%  (9.3-23.5) | 7.5%  (2.1-23.8) | 2.3%  (0.2-20.0) | 2.7%  (0.6-11.4) | 8.1%  (6.2-10.6) | 0.044 |
| Epididymitis | 0.7%  (0.1-3.8) | 0.0%  (-) | 1.1%  (0.2-6.5) | 0.0%  (-) | 0.0%  (-) | 2.3%  (0.2-20.0) | 0.0%  (-) | 0.5%  (0.2-1.3) | 0.745 |
| HIV | 2.1%  (0.8-5.2) | 3.6%  (1.2-10.0) | 3.3%  (1.4-7.6) | 6.3%  (3.5-11.2) | 2.5%  (0.4-13.3) | 9.3%  (2.6-28.5) | 5.5%  (1.7-15.9) | 3.9%  (2.8-5.4) | 0.225 |
| Cannot remember which STI | 12.4%  (10.0-15.3) | 11.6%  (5.5-22.8) | 15.6%  (6.7-32.2) | 10.3%  (5.9-17.4) | 17.5%  (11.6-25.5) | 11.6%  (5.3-23.5) | 13.7%  (5.4-30.5) | 12.7%  (10.2-15.6) | 0.804 |
| **Of those reporting diagnosis/treatment of bacterial STI(s) or trichomoniasis, at last STI episode^c^**  *Denominator:* | *112* | *64* | *52* | *78* | *15* | *21* | *41* | *383* |  |
| At that time, did the clinic staff advise you to inform your sexual partners to test for STIs/come to clinic? |  |  |  |  |  |  |  |  | 0.213 |
| No | 10.8%  (7.7-15.1) | 9.7%  (4.2-20.8) | 5.1%  (2.4-10.5) | 6.4%  (2.7-14.4) | 9.5%  (2.7-28.9) | 12.5%  (4.9-28.5) | 8.5%  (3.1-21.2) | 8.9%  (6.3-12.2) |  |
| Yes | 73.9%  (64.3-81.6) | 84.7%  (71.2-92.6) | 83.1%  (76.7-88.0) | 80.9%  (71.6-87.6) | 81.0%  (59.7-92.4) | 66.7%  (56.3-75.6) | 85.1%  (72.7-92.5) | 79.1%  (74.6-83.0) |  |
| Can't remember | 15.3%  (9.8-23.1) | 5.6%  (2.1-13.8) | 11.9%  (7.6-18.0) | 12.8%  (6.8-22.6) | 9.5%  (1.8-37.1) | 20.8%  (16.6-25.8) | 6.4%  (2.6-15.0) | 12.0%  (8.4-17.0) |  |
| At that time, did you inform your sexual partners to test/take treatment for STIs? |  |  |  |  |  |  |  |  | 0.044 |
| No, I didn't tell any partners | 20.4%  (15.1-27.0) | 2.8%  (0.9-8.7) | 5.0%  (1.3-17.7) | 8.5%  (4.7-14.9) | 23.8%  (11.9-41.9) | 4.2%  (0.5-26.4) | 4.3%  (1.0-17.0) | 11.2%  (8.5-14.5) |  |
| Yes, I told ALL my partners | 56.1%  (44.3-67.2) | 68.1%  (58.9-76.0) | 80.0%  (71.5-86.5) | 77.7%  (69.6-84.1) | 57.1%  (33.0-78.3) | 62.5%  (38.4-81.7) | 76.6%  (61.9-86.8) | 67.6%  (61.2-73.4) |  |
| Can't remember | 8.3%  (4.3-15.5) | 8.3%  (5.9-11.6) | 8.3%  (1.9-29.7) | 8.5%  (2.8-23.0) | 4.8%  (0.5-33.8) | 8.3%  (2.2-26.8) | 8.5%  (3.8-18.1) | 8.2%  (5.6-11.8) |  |
| Yes, I told SOME of my partners | 15.3%  (7.2-29.4) | 20.8%  (12.3-33.0) | 6.7%  (2.7-15.7) | 5.3%  (2.2-12.4) | 14.3%  (4.6-36.7) | 25.0%  (14.5-39.6) | 10.6%  (4.1-25.0) | 13.1%  (9.1-18.3) |  |
| **Of those diagnosed with/treated bacterial STI/trichomoniasis,^c^**  **AND who informed some/all partners to test/come to the clinic**  *Denominator:* | *112* | *64* | *52* | *78* | *15* | *21* | *41* | *383* |  |
| At that time, how did you inform you sexual partners to test for STIs/come to clinic?^b^ |  |  |  |  |  |  |  |  |  |
| In person | 58.9%  (49.2-68.0) | 53.1%  (43.9-62.1) | 76.9%  (60.7-87.8) | 65.4%  (50.9-77.5) | 60.0%  (28.0-85.3) | 61.9%  (36.3-82.2) | 68.3%  (51.5-81.4) | 62.9%  (55.0-70.2) | 0.232 |
| Via a text message | 38.4%  (27.3-50.9) | 43.8%  (27.5-61.4) | 21.2%  (12.2-34.1) | 19.2%  (12.6-28.1) | 26.7%  (8.4-59.2) | 28.6%  (10.3-58.3) | 29.3%  (19.3-41.8) | 31.1%  (25.4-37.4) | 0.092 |
| Via an email | 1.8%  (0.4-7.5) | 1.6%  (0.2-13.2) | 0.0%  (-) | 0.0%  (-) | 0.0%  (-) | 0.0%  (-) | 0.0%  (-) | 0.8%  (0.3-2.0) | 0.764 |
| Via telephone | 17.9%  (13.6-23.1) | 21.9%  (10.5-40.1) | 25.0%  (16.0-36.8) | 33.3%  (26.0-41.6) | 33.3%  (18.8-51.9) | 23.8%  (8.2-52.1) | 29.3%  (12.9-53.7) | 24.8%  (21.4-28.5) | 0.348 |
| Via social media | 6.3%  (2.4-15.6) | 9.4%  (3.3-23.8) | 3.8%  (0.9-14.7) | 2.6%  (0.6-10.3) | 6.7%  (0.6-45.5) | 4.8%  (0.6-30.9) | 0.0%  (-) | 5.0%  (2.4-10.2) | 0.416 |
| Via a friend | 0.9%  (0.1-6.3) | 0.0%  (-) | 0.0%  (-) | 1.3%  (0.2-9.8) | 0.0%  (-) | 0.0%  (-) | 0.0%  (-) | 0.5%  (0.1-2.0) | 0.889 |
| Via a clinic health adviser/clinic staff | 0.9%  (0.1-8.1) | 0.0%  (-) | 0.0%  (-) | 1.3%  (0.1-10.6) | 0.0%  (-) | 4.8%  (1.1-18.1) | 0.0%  (-) | 0.8%  (0.3-2.2) | 0.661 |
| Other | 0.9%  (0.1-8.1) | 0.0%  (-) | 0.0%  (-) | 1.3%  (0.1-10.6) | 0.0%  (-) | 0.0%  (-) | 0.0%  (-) | 0.5%  (0.1-2.2) | 0.889 |
| **Of those diagnosed with/treated bacterial STI/trichomoniasis,**  **AND who did NOT inform ALL of their partners to test/attend clinic (informed none, or informed some)^b,c,d^** |  |  |  |  |  |  |  |  |  |
| *Denominator:* | 56 | 17 | 7 | 13 | 8 | 7 | 7 | 115 |  |
| At that time, why did you not inform (some of) your sexual partners to test for the infection/come to the clinic? | n | n | n | n | n | n | n | n |  |
| My partner(s) lives outside the UK | 4 | 4 | 0 | 0 | 0 | 1 | 1 | 10 |  |
| I was embarrassed to tell my partner(s) about the infection | 10 | 2 | 1 | 4 | 1 | 0 | 2 | 20 |  |
| I was scared of telling my partner(s) about the infection | 5 | 0 | 2 | 3 | 1 | 0 | 0 | 11 |  |
| I was worried that my partner(s) would leave me | 1 | 0 | 1 | 4 | 1 | 0 | 0 | 7 |  |
| I did not have contact details of my partner(s) | 29 | 13 | 0 | 1 | 5 | 5 | 4 | 57 |  |
| I was not too concerned about telling my main partner | 2 | 1 | 2 | 1 | 0 | 0 | 0 | 6 |  |
| I was not too concerned about telling my casual / one-off partner(s) | 9 | 4 | 2 | 1 | 2 | 1 | 1 | 20 |  |
| Other | 12 | 2 | 2 | 5 | 0 | 1 | 2 | 24 |  |

^a^STIs listed included: chlamydia, gonorrhoea, genital warts (venereal warts), syphilis, *Trichomonas vaginalis* (Trich, TV), herpes (genital herpes), hepatitis B, NSU/NGU (non-specific urethritis/non-gonococcal urethritis), epididymitis, HIV. Women selecting NSU/NGU or epididymitis may have selected this option if they were treated as sexual contacts of men with these diagnoses.

^b^Multiple responses were permitted.

^c^Chlamydia, gonorrhoea, syphilis, Trich, NSU/NGU, epididymitis (PID was not in the questionnaire). We only included bacterial STIs and trichomoniasis in the questions about partner notification (PN) because they are acute infections for which PN should be addressed when diagnosed/treated. (Viral STIs may be may be chronic and require repeat treatments, and therefore there would not necessarily have been a routine PN discussion when last treated; furthermore, PN is not routinely advised for some viral STIs, e.g. warts, herpes).[21]

^d^Numbers are provided in place of percentages, because of low denominators.
