# Supplementary material for: Pathways to, and use of, sexual healthcare among Black Caribbean sexual health clinic attendees in England: evidence from cross-sectional bio-behavioural surveys
Source: BMC Health Serv Res. 2019 Sep 18;19:668. doi: 10.1186/s12913-019-4396-3 (PMC6749649; doi:10.1186/s12913-019-4396-3)
Supplement: Supplementary file 6 — Text of the online survey (PDF 740 kb) [file 12913_2019_4396_MOESM6_ESM.pdf]

RiiSH

Please type the unique passcode given on the invitation card in the box below:

____

Consent Statement

You have been given an invitation card to participate in the RiiSH survey because everyone aged 15 years and above coming to this sexual health clinic is being approached to participate in this survey. Your participation is voluntary. This study is being conducted by the Centre for Sexual Health and HIV Research, University College London, London in collaboration with Public Health England.

What is the survey about?
This survey asks about your sexual behaviour, factors that influence your sexual behaviour and precautions you take, and your use of health services including STI clinics. The survey is CONFIDENTIAL – you will not be asked for your name or address or any identifiable data or means of contacting you in the future. Please answer all questions honestly. Please complete this survey ONLY ONCE. The survey results will help us understand the reasons for sexual inequalities in health and develop interventions to improve healthcare.

What will happen if you decide to take part?

It will take approximately 10-15 minutes to complete the survey. You will not lose your place in the clinic queue because you are doing the survey. Once you complete the survey and submit your answers, the data will be stored on a secure server and will be treated confidentially. The clinic staff will not be able to see your survey responses. If you agree, we will link your survey answers to existing information on STI tests and diagnoses that is routinely collected by Public Health England from sexual health clinics in England. We will link your survey answers to the routinely collected data using the unique passcode you typed at the start of the survey and your clinic number that will be given to us by the clinic.

Once the data are linked the unique passcode and your clinic number will be removed. Only anonymous data will be analysed and reported on. If you do not agree to the linkage, you can still participate in the survey but we will not link your answers to the routinely collected data.

Thank you very much for taking the time to consider taking part in this study.

Please tick the appropriate box to indicate whether you agree to participate in this survey. If you tick the box 'I do not agree to participate', you will exit the survey.

- ❑ I have read and understood the participant information sheet (v2: 08.02/2016) and I agree to participate in the survey
- ❑ I do not agree to participate in the survey

Please tick the appropriate box to indicate whether you agree to link your survey answers to existing routinely collected data on STI diagnosis and testing. If you tick the box ‘I agree to the linkage’ the linked data will be completely and irreversibly anonymised before being analysed.

- ❑ I agree to the linkage
- ❑ I do not agree to the linkage

First we will ask you a few questions about yourself.

How old are you?

- ❑ under 15
- ❑ 15
- ❑ 16
- ❑ 17
- ❑ 18

- ❑ 19
- ❑ 20
- ❑ 21
- ❑ 22
- ❑ 23
- ❑ 24
- ❑ 25
- ❑ 26
- ❑ 27
- ❑ 28
- ❑ 29
- ❑ 30
- ❑ 31
- ❑ 32
- ❑ 33
- ❑ 34
- ❑ 35
- ❑ 36
- ❑ 37
- ❑ 38
- ❑ 39
- ❑ 40
- ❑ 41
- ❑ 42
- ❑ 43
- ❑ 44
- ❑ 45
- ❑ 46
- ❑ 47
- ❑ 48
- ❑ 49
- ❑ 50
- ❑ 51
- ❑ 52
- ❑ 53
- ❑ 54
- ❑ 55
- ❑ 56
- ❑ 57
- ❑ 58

- ❑ 59
- ❑ 60
- ❑ 61
- ❑ 62
- ❑ 63
- ❑ 64
- ❑ 65
- ❑ 66
- ❑ 67
- ❑ 68
- ❑ 69
- ❑ 70
- ❑ 71
- ❑ 72
- ❑ 73
- ❑ 74
- ❑ 75
- ❑ 76
- ❑ 77
- ❑ 78
- ❑ 79
- ❑ 80
- ❑ Over 80

How do you identify yourself?

- ❑ Male
- ❑ Female
- ❑ Trans Male
- ❑ Trans Female
- ❑ Genderqueer
- ❑ Non-binary
- ❑ Indeterminate (intersex)
- ❑ Other

In the last 12 months have had sex with you:

- ❑ Men
- ❑ Women
- ❑ Both men and women
- ❑ No one

Which sexual health clinic invited you to take part in this survey? Please read the following options carefully:

- ❑ Ambrose Clinic (Royal London Hospital, Mount Terrace)
- ❑ Archway Centre for Sexual Health and Contraceptive Care (Holloway road, Archway)
- ❑ Boots City Centre (Birmingham)
- ❑ Burrell Street Clinic (Guys and St Thomas Hospital, Burrell Street)
- ❑ Camberwell Sexual Health Centre (Kings College Hospital, Denmark Hill)
- ❑ Clifton Centre (Homerton University Hospital, Homerton)
- ❑ Croydon Sexual Health Centre (Croydon University Hospital, Croydon)
- ❑ Erdington Umbrella Clinic (Birmingham)
- ❑ Ivy Sexual Health Clinic (St Leonard's Nuttall Street)
- ❑ Jefferiss Wing Centre for Sexual Health (St Mary's Hospital, Praed Street)
- ❑ Lloyds clinic (Guys and St Thomas Hospital, Great Maze Pond)
- ❑ Northwick Park Sexual Health Clinic (Northwick Park Hospital, Harrow)
- ❑ Patrick Clements GUM Centre (Central Middlesex Hospital, Park Royal)
- ❑ Streatham Hill Centre for Sexual Health (Streatham Hill)
- ❑ The Courtyard Clinic (St George's Hospital, Tooting)
- ❑ Umbrella Sexual Health Services (Whittall Street, Birmingham)
- ❑ Waldron Sexual Health Clinic (Waldron Health Centre, New Cross)
- ❑ 10 Hammersmith Broadway
- ❑ Other

Please specify

____

____

On which date (**DD/MM/YY**) did you attend the clinic?

____

Where did you do the survey?

- ❑ In the clinic
- ❑ At home on the same day I attended the clinic
- ❑ At home but ***not*** on the same day I attended the clinic

Where were you born?

- ❑ In the UK
- ❑ Outside the UK

In which country were you born? (If it does not exist anymore, please select the country that best applies.)

- ❑ Afghanistan
- ❑ Albania
- ❑ Algeria
- ❑ Andorra
- ❑ Angola
- ❑ Antigua and Barbuda
- ❑ Argentina
- ❑ Armenia
- ❑ Australia
- ❑ Austria
- ❑ Azerbaijan
- ❑ Bahamas
- ❑ Bahrain
- ❑ Bangladesh
- ❑ Barbados
- ❑ Belarus
- ❑ Belgium
- ❑ Belize
- ❑ Benin
- ❑ Bhutan
- ❑ Bolivia
- ❑ Bosnia and Herzegovina
- ❑ Botswana
- ❑ Brazil
- ❑ Brunei
- ❑ Bulgaria
- ❑ Burkina Faso
- ❑ Burundi
- ❑ Cambodia
- ❑ Cameroon
- ❑ Canada
- ❑ Cape Verde/Cabo Verde
- ❑ Central African Republic
- ❑ Chad
- ❑ Chile
- ❑ China
- ❑ Colombia
- ❑ Comoros
- ❑ Congo - Kinshasa
- ❑ Congo - Brazzaville

- ❑ Costa Rica
- ❑ Croatia
- ❑ Cuba
- ❑ Cyprus
- ❑ Czech Republic
- ❑ Denmark
- ❑ Djibouti
- ❑ Dominica
- ❑ Dominican Republic
- ❑ Ecuador
- ❑ Egypt
- ❑ El Salvador
- ❑ Equatorial Guinea
- ❑ Eritrea
- ❑ Estonia
- ❑ Ethiopia
- ❑ Fiji
- ❑ Finland
- ❑ France
- ❑ France, Overseas Departments, Territories and Collectivities
- ❑ Gabon
- ❑ Gambia
- ❑ Georgia
- ❑ Germany
- ❑ Ghana
- ❑ Greece
- ❑ Greenland
- ❑ Grenada
- ❑ Guatemala
- ❑ Guinea
- ❑ Guinea-Bissau
- ❑ Guyana
- ❑ Haiti
- ❑ Honduras
- ❑ Hungary
- ❑ Iceland
- ❑ India
- ❑ Indonesia
- ❑ Iran
- ❑ Iraq

- ❑ Ireland (Republic of)
- ❑ Israel
- ❑ Italy
- ❑ Ivory Coast
- ❑ Jamaica
- ❑ Japan
- ❑ Jordan
- ❑ Kazakhstan
- ❑ Kenya
- ❑ Kiribati
- ❑ Korea, North
- ❑ Korea, South
- ❑ Kosovo
- ❑ Kuwait
- ❑ Kyrgyzstan
- ❑ Laos
- ❑ Latvia
- ❑ Lebanon
- ❑ Lesotho
- ❑ Liberia
- ❑ Libya
- ❑ Liechtenstein
- ❑ Lithuania
- ❑ Luxembourg
- ❑ Macedonia (Former Yugoslav Republic of)
- ❑ Madagascar
- ❑ Malawi
- ❑ Malaysia
- ❑ Maldives
- ❑ Mali
- ❑ Malta
- ❑ Marshall Islands
- ❑ Mauritania
- ❑ Mauritius
- ❑ Mexico
- ❑ Micronesia
- ❑ Moldova
- ❑ Monaco
- ❑ Mongolia

- ❑ Montenegro
- ❑ Morocco
- ❑ Mozambique
- ❑ Myanmar
- ❑ Namibia
- ❑ Nauru
- ❑ Nepal
- ❑ Netherlands
- ❑ New Zealand
- ❑ Nicaragua
- ❑ Niger
- ❑ Nigeria
- ❑ Norway
- ❑ Oman
- ❑ Pakistan
- ❑ Palau
- ❑ Palestine
- ❑ Panama
- ❑ Papua New Guinea
- ❑ Paraguay
- ❑ Peru
- ❑ Philippines
- ❑ Poland
- ❑ Portugal
- ❑ Puerto Rico
- ❑ Qatar
- ❑ Romania
- ❑ Russia (Russian Federation)
- ❑ Rwanda
- ❑ Saint Kitts and Nevis
- ❑ Saint Lucia
- ❑ Saint Vincent and the Grenadines
- ❑ Samoa
- ❑ San Marino
- ❑ Sao Tome and Principe
- ❑ Saudi Arabia
- ❑ Senegal
- ❑ Serbia
- ❑ Seychelles

- ❑ Sierra Leone
- ❑ Singapore
- ❑ Slovakia
- ❑ Slovenia
- ❑ Solomon Islands
- ❑ Somalia
- ❑ South Africa
- ❑ Spain
- ❑ Sri Lanka
- ❑ Sudan
- ❑ Republic of South Sudan
- ❑ Suriname
- ❑ Swaziland
- ❑ Sweden
- ❑ Switzerland
- ❑ Syria
- ❑ Taiwan
- ❑ Tajikistan
- ❑ Tanzania
- ❑ Thailand
- ❑ Timor-Leste
- ❑ Togo
- ❑ Tonga
- ❑ Trinidad and Tobago
- ❑ Tunisia
- ❑ Turkey
- ❑ Turkish Republic of Northern Cyprus
- ❑ Turkmenistan
- ❑ Tuvalu
- ❑ Uganda
- ❑ Ukraine
- ❑ United Arab Emirates
- ❑ United States of America
- ❑ Uruguay
- ❑ Uzbekistan
- ❑ Vanuatu
- ❑ Vatican City
- ❑ Venezuela
- ❑ Vietnam

- ❑ Western Sahara
- ❑ Yemen
- ❑ Zambia
- ❑ Zimbabwe

How many years have you been living in the UK?

- ❑ Less than 1 year
- ❑ 1
- ❑ 2
- ❑ 3
- ❑ 4
- ❑ 5
- ❑ 6
- ❑ 7
- ❑ 8
- ❑ 9
- ❑ 10
- ❑ 11
- ❑ 12
- ❑ 13
- ❑ 14
- ❑ 15
- ❑ 16
- ❑ 17
- ❑ 18
- ❑ 19
- ❑ 20
- ❑ 21
- ❑ 22
- ❑ 23
- ❑ 24
- ❑ 25
- ❑ 26
- ❑ 27
- ❑ 28
- ❑ 29
- ❑ 30
- ❑ 31

- ❑ 32
- ❑ 33
- ❑ 34
- ❑ 35
- ❑ 36
- ❑ 37
- ❑ 38
- ❑ 39
- ❑ 40
- ❑ 41
- ❑ 42
- ❑ 43
- ❑ 44
- ❑ 45
- ❑ 46
- ❑ 47
- ❑ 48
- ❑ 49
- ❑ 50
- ❑ 51
- ❑ 52
- ❑ 53
- ❑ 54
- ❑ 55
- ❑ 56
- ❑ 57
- ❑ 58
- ❑ 59
- ❑ 60
- ❑ 61
- ❑ 62
- ❑ 63
- ❑ 64
- ❑ 65
- ❑ 66
- ❑ 67
- ❑ 68
- ❑ 69

- ❑ 70
- ❑ 71
- ❑ 72
- ❑ 73
- ❑ 74
- ❑ 75
- ❑ 76
- ❑ 77
- ❑ 78
- ❑ 79
- ❑ 80
- ❑ More than 80

Which of the following best describes your ethnic group?

- ❑ White: English/Welsh/Scottish/Northern Irish/British
- ❑ White Irish
- ❑ Any other white background
- ❑ Black/ Black British: African
- ❑ Black/ Black British: Caribbean
- ❑ Black/ Black British:Any other Black background
- ❑ Asian/ Asian British: Indian
- ❑ Asian/Asian British: Pakistani
- ❑ Asian/Asian British: Bangladeshi
- ❑ Asian/Asian British: Chinese
- ❑ Asian/Asian British: Any other Asian background
- ❑ Mixed: White and Black Caribbean
- ❑ Mixed: White and Black African
- ❑ Mixed: White and Asian
- ❑ Mixed: any other mixed/multiple background
- ❑ Arab
- ❑ Any other ethnic group
- ❑ Decline to answer

Please specify

Please specify

____

Please specify

____

Please specify

____

Please specify

____

Where was your natural/biological FATHER born?

- ☐ In the UK
- ☐ Outside the UK
- ☐ Don't know

In which country was your FATHER born? (If it does not exist anymore, please select the country that best applies.)

- ☐ Afghanistan
- ☐ Albania
- ☐ Algeria
- ☐ Andorra
- ☐ Angola
- ☐ Antigua and Barbuda
- ☐ Argentina
- ☐ Armenia
- ☐ Australia
- ☐ Austria
- ☐ Azerbaijan
- ☐ Bahamas
- ☐ Bahrain
- ☐ Bangladesh
- ☐ Barbados
- ☐ Belarus

- ❑ Belgium
- ❑ Belize
- ❑ Benin
- ❑ Bhutan
- ❑ Bolivia
- ❑ Bosnia and Herzegovina
- ❑ Botswana
- ❑ Brazil
- ❑ Brunei
- ❑ Bulgaria
- ❑ Burkina Faso
- ❑ Burundi
- ❑ Cambodia
- ❑ Cameroon
- ❑ Canada
- ❑ Cape Verde/Cabo Verde
- ❑ Central African Republic
- ❑ Chad
- ❑ Chile
- ❑ China
- ❑ Colombia
- ❑ Comoros
- ❑ Congo - Kinshasa
- ❑ Congo - Brazzaville
- ❑ Costa Rica
- ❑ Croatia
- ❑ Cuba
- ❑ Cyprus
- ❑ Czech Republic
- ❑ Denmark
- ❑ Djibouti
- ❑ Dominica
- ❑ Dominican Republic
- ❑ Ecuador
- ❑ Egypt
- ❑ El Salvador
- ❑ Equatorial Guinea
- ❑ Eritrea

- ❑ Estonia
- ❑ Ethiopia
- ❑ Fiji
- ❑ Finland
- ❑ France
- ❑ France, Overseas Departments, Territories and Collectivities
- ❑ Gabon
- ❑ Gambia
- ❑ Georgia
- ❑ Germany
- ❑ Ghana
- ❑ Greece
- ❑ Greenland
- ❑ Grenada
- ❑ Guatemala
- ❑ Guinea
- ❑ Guinea-Bissau
- ❑ Guyana
- ❑ Haiti
- ❑ Honduras
- ❑ Hungary
- ❑ Iceland
- ❑ India
- ❑ Indonesia
- ❑ Iran
- ❑ Iraq
- ❑ Ireland (Republic of)
- ❑ Israel
- ❑ Italy
- ❑ Ivory Coast
- ❑ Jamaica
- ❑ Japan
- ❑ Jordan
- ❑ Kazakhstan
- ❑ Kenya
- ❑ Kiribati
- ❑ Korea, North
- ❑ Korea, South

- ❑ Kosovo
- ❑ Kuwait
- ❑ Kyrgyzstan
- ❑ Laos
- ❑ Latvia
- ❑ Lebanon
- ❑ Lesotho
- ❑ Liberia
- ❑ Libya
- ❑ Liechtenstein
- ❑ Lithuania
- ❑ Luxembourg
- ❑ Macedonia (Former Yugoslav Republic of)
- ❑ Madagascar
- ❑ Malawi
- ❑ Malaysia
- ❑ Maldives
- ❑ Mali
- ❑ Malta
- ❑ Marshall Islands
- ❑ Mauritania
- ❑ Mauritius
- ❑ Mexico
- ❑ Micronesia
- ❑ Moldova
- ❑ Monaco
- ❑ Mongolia
- ❑ Montenegro
- ❑ Morocco
- ❑ Mozambique
- ❑ Myanmar
- ❑ Namibia
- ❑ Nauru
- ❑ Nepal
- ❑ Netherlands
- ❑ New Zealand
- ❑ Nicaragua
- ❑ Niger

- ❑ Nigeria
- ❑ Norway
- ❑ Oman
- ❑ Pakistan
- ❑ Palau
- ❑ Palestine
- ❑ Panama
- ❑ Papua New Guinea
- ❑ Paraguay
- ❑ Peru
- ❑ Philippines
- ❑ Poland
- ❑ Portugal
- ❑ Puerto Rico
- ❑ Qatar
- ❑ Romania
- ❑ Russia (Russian Federation)
- ❑ Rwanda
- ❑ Saint Kitts and Nevis
- ❑ Saint Lucia
- ❑ Saint Vincent and the Grenadines
- ❑ Samoa
- ❑ San Marino
- ❑ Sao Tome and Principe
- ❑ Saudi Arabia
- ❑ Senegal
- ❑ Serbia
- ❑ Seychelles
- ❑ Sierra Leone
- ❑ Singapore
- ❑ Slovakia
- ❑ Slovenia
- ❑ Solomon Islands
- ❑ Somalia
- ❑ South Africa
- ❑ Spain
- ❑ Sri Lanka
- ❑ Sudan

- ❑ Republic of South Sudan
- ❑ Suriname
- ❑ Swaziland
- ❑ Sweden
- ❑ Switzerland
- ❑ Syria
- ❑ Taiwan
- ❑ Tajikistan
- ❑ Tanzania
- ❑ Thailand
- ❑ Timor-Leste
- ❑ Togo
- ❑ Tonga
- ❑ Trinidad and Tobago
- ❑ Tunisia
- ❑ Turkey
- ❑ Turkish Republic of Northern Cyprus
- ❑ Turkmenistan
- ❑ Tuvalu
- ❑ Uganda
- ❑ Ukraine
- ❑ United Arab Emirates
- ❑ United States of America
- ❑ Uruguay
- ❑ Uzbekistan
- ❑ Vanuatu
- ❑ Vatican City
- ❑ Venezuela
- ❑ Vietnam
- ❑ Western Sahara
- ❑ Yemen
- ❑ Zambia
- ❑ Zimbabwe

Which of the following best describes your FATHER's ethnic group?

- ❑ White: English/Welsh/Scottish/Northern Irish/British
- ❑ White Irish
- ❑ Any other white background
- ❑ Black/ Black British: African
- ❑ Black/ Black British: Caribbean
- ❑ Black/ Black British:Any other Black background
- ❑ Asian/ Asian British: Indian
- ❑ Asian/Asian British: Pakistani
- ❑ Asian/Asian British: Bangladeshi
- ❑ Asian/Asian British: Chinese
- ❑ Asian/Asian British: Any other Asian background
- ❑ Mixed: White and Black Caribbean
- ❑ Mixed: White and Black African
- ❑ Mixed: White and Asian
- ❑ Mixed: any other mixed/multiple background
- ❑ Arab
- ❑ Any other ethnic group
- ❑ Decline to answer

Please specify

____

Please specify

____

Please specify

____

Please specify

____

Please specify

____

In which country was your natural/biological MOTHER born?

- ❑ In the UK
- ❑ Outside the UK
- ❑ Don't know

In which country was your MOTHER born? (If it does not exist anymore, please select the country that best applies.)

- ❑ Afghanistan
- ❑ Albania
- ❑ Algeria
- ❑ Andorra
- ❑ Angola
- ❑ Antigua and Barbuda
- ❑ Argentina
- ❑ Armenia
- ❑ Australia
- ❑ Austria
- ❑ Azerbaijan
- ❑ Bahamas
- ❑ Bahrain
- ❑ Bangladesh
- ❑ Barbados
- ❑ Belarus
- ❑ Belgium
- ❑ Belize
- ❑ Benin
- ❑ Bhutan
- ❑ Bolivia
- ❑ Bosnia and Herzegovina
- ❑ Botswana
- ❑ Brazil
- ❑ Brunei
- ❑ Bulgaria
- ❑ Burkina Faso
- ❑ Burundi
- ❑ Cambodia

- ❑ Cameroon
- ❑ Canada
- ❑ Cape Verde/Cabo Verde
- ❑ Central African Republic
- ❑ Chad
- ❑ Chile
- ❑ China
- ❑ Colombia
- ❑ Comoros
- ❑ Congo - Kinshasa
- ❑ Congo - Brazzaville
- ❑ Costa Rica
- ❑ Croatia
- ❑ Cuba
- ❑ Cyprus
- ❑ Czech Republic
- ❑ Denmark
- ❑ Djibouti
- ❑ Dominica
- ❑ Dominican Republic
- ❑ Ecuador
- ❑ Egypt
- ❑ El Salvador
- ❑ Equatorial Guinea
- ❑ Eritrea
- ❑ Estonia
- ❑ Ethiopia
- ❑ Fiji
- ❑ Finland
- ❑ France
- ❑ France, Overseas Departments, Territories and Collectivities
- ❑ Gabon
- ❑ Gambia
- ❑ Georgia
- ❑ Germany
- ❑ Ghana
- ❑ Greece
- ❑ Greenland

- ❑ Grenada
- ❑ Guatemala
- ❑ Guinea
- ❑ Guinea-Bissau
- ❑ Guyana
- ❑ Haiti
- ❑ Honduras
- ❑ Hungary
- ❑ Iceland
- ❑ India
- ❑ Indonesia
- ❑ Iran
- ❑ Iraq
- ❑ Ireland (Republic of)
- ❑ Israel
- ❑ Italy
- ❑ Ivory Coast
- ❑ Jamaica
- ❑ Japan
- ❑ Jordan
- ❑ Kazakhstan
- ❑ Kenya
- ❑ Kiribati
- ❑ Korea, North
- ❑ Korea, South
- ❑ Kosovo
- ❑ Kuwait
- ❑ Kyrgyzstan
- ❑ Laos
- ❑ Latvia
- ❑ Lebanon
- ❑ Lesotho
- ❑ Liberia
- ❑ Libya
- ❑ Liechtenstein
- ❑ Lithuania
- ❑ Luxembourg
- ❑ Macedonia (Former Yugoslav Republic of)

- ❑ Madagascar
- ❑ Malawi
- ❑ Malaysia
- ❑ Maldives
- ❑ Mali
- ❑ Malta
- ❑ Marshall Islands
- ❑ Mauritania
- ❑ Mauritius
- ❑ Mexico
- ❑ Micronesia
- ❑ Moldova
- ❑ Monaco
- ❑ Mongolia
- ❑ Montenegro
- ❑ Morocco
- ❑ Mozambique
- ❑ Myanmar
- ❑ Namibia
- ❑ Nauru
- ❑ Nepal
- ❑ Netherlands
- ❑ New Zealand
- ❑ Nicaragua
- ❑ Niger
- ❑ Nigeria
- ❑ Norway
- ❑ Oman
- ❑ Pakistan
- ❑ Palau
- ❑ Palestine
- ❑ Panama
- ❑ Papua New Guinea
- ❑ Paraguay
- ❑ Peru
- ❑ Philippines
- ❑ Poland
- ❑ Portugal

- ❑ Puerto Rico
- ❑ Qatar
- ❑ Romania
- ❑ Russia (Russian Federation)
- ❑ Rwanda
- ❑ Saint Kitts and Nevis
- ❑ Saint Lucia
- ❑ Saint Vincent and the Grenadines
- ❑ Samoa
- ❑ San Marino
- ❑ Sao Tome and Principe
- ❑ Saudi Arabia
- ❑ Senegal
- ❑ Serbia
- ❑ Seychelles
- ❑ Sierra Leone
- ❑ Singapore
- ❑ Slovakia
- ❑ Slovenia
- ❑ Solomon Islands
- ❑ Somalia
- ❑ South Africa
- ❑ Spain
- ❑ Sri Lanka
- ❑ Sudan
- ❑ Republic of South Sudan
- ❑ Suriname
- ❑ Swaziland
- ❑ Sweden
- ❑ Switzerland
- ❑ Syria
- ❑ Taiwan
- ❑ Tajikistan
- ❑ Tanzania
- ❑ Thailand
- ❑ Timor-Leste
- ❑ Togo
- ❑ Tonga

- ❑ Trinidad and Tobago
- ❑ Tunisia
- ❑ Turkey
- ❑ Turkish Republic of Northern Cyprus
- ❑ Turkmenistan
- ❑ Tuvalu
- ❑ Uganda
- ❑ Ukraine
- ❑ United Arab Emirates
- ❑ United States of America
- ❑ Uruguay
- ❑ Uzbekistan
- ❑ Vanuatu
- ❑ Vatican City
- ❑ Venezuela
- ❑ Vietnam
- ❑ Western Sahara
- ❑ Yemen
- ❑ Zambia
- ❑ Zimbabwe

Which of the following best describes your MOTHER's ethnic group?

- ❑ White: English/Welsh/Scottish/Northern Irish/British
- ❑ White Irish
- ❑ Any other white background
- ❑ Black/ Black British: African
- ❑ Black/ Black British: Caribbean
- ❑ Black/ Black British:Any other Black background
- ❑ Asian/ Asian British: Indian
- ❑ Asian/Asian British: Pakistani
- ❑ Asian/Asian British: Bangladeshi
- ❑ Asian/Asian British: Chinese
- ❑ Asian/Asian British: Any other Asian background
- ❑ Mixed: White and Black Caribbean
- ❑ Mixed: White and Black African
- ❑ Mixed: White and Asian

- ❑ Mixed: any other mixed/multiple background
- ❑ Arab
- ❑ Any other ethnic group
- ❑ Decline to answer

Please specify ____

Please specify ____

Please specify ____

Please specify ____

Please specify ____

What is your HIGHEST educational qualification so far?

- ❑ No educational qualifications
- ❑ GCSEs/CSEs/O-Levels or equivalent/CSEC
- ❑ A-Levels/AS level/Higher school certificate/CAPE
- ❑ GNNQ/NVQ levels 1-3
- ❑ NVQ levels 4-5/HNC/HND
- ❑ Degree or higher degree
- ❑ Other qualifications gained inside the UK (e.g. City and Guilds)

Which of the following best describes your current occupation? (Tick ALL that apply)

- ❑ Employed full-time
- ❑ Employed part-time
- ❑ Self-employed
- ❑ Unemployed
- ❑ Student
- ❑ Retired

- ❑ Long-term sick leave / medically retired
- ❑ Other

Please specify

____

What is your sexual orientation?

- ❑ Straight / heterosexual
- ❑ Bisexual
- ❑ Gay / homosexual / lesbian
- ❑ Rather not say

Currently you: (Tick ALL that apply)

- ❑ have a partner with whom you are married or in a civil partnership
- ❑ have partner(s) you are committed to but with whom you are not married or in a civil partrnership
- ❑ have partner(s) you are not committed to but you have sex with regularly
- ❑ have partner(s) with whom you have sex but not regularly
- ❑ have one-off sex partner(s)
- ❑ don't have partner(s) currently

Are you currently living with a partner?

- ❑ Yes, I live with a partner
- ❑ No, I don't live with a partner

The following questions are about your risk of getting sexually transmitted infections.

Which of the following sexually transmitted infections have you heard of? (Tick ALL that apply)

- ❑ None
- ❑ Chlamydia
- ❑ Gonorrhoea
- ❑ Genital Warts (venereal warts)
- ❑ Syphilis
- ❑ Trichomonas vaginalis (Trich, TV)

- ❑ Herpes (genital herpes)
- ❑ Hepatitis A
- ❑ Hepatitis B
- ❑ Hepatitis C
- ❑ HIV
- ❑ Other

Please specify

____

Thinking about your current sexual lifestyle, which of the following STIs do you think you may be at risk of getting? (Tick ALL that apply)

- ❑ I don’t think I am at risk of getting any STI
- ❑ Chlamydia
- ❑ Gonorrhoea
- ❑ Genital Warts (venereal warts)
- ❑ Syphilis
- ❑ Trichomonas vaginalis (Trich, TV)
- ❑ Herpes (genital herpes)
- ❑ Hepatitis A
- ❑ Hepatitis B
- ❑ Hepatitis C
- ❑ HIV
- ❑ Don't know
- ❑ Other

Please specify

____

The following questions are about your sexual behaviour and partnerships.

How old were you the first time you had any kind of sex with someone?

- ❑ Less than 8
- ❑ 8
- ❑ 9

- ❑ 10
- ❑ 11
- ❑ 12
- ❑ 13
- ❑ 14
- ❑ 15
- ❑ 16
- ❑ 17
- ❑ 18
- ❑ 19
- ❑ 20
- ❑ 21
- ❑ 22
- ❑ 23
- ❑ 24
- ❑ 25
- ❑ 26
- ❑ 27
- ❑ 28
- ❑ 29
- ❑ 30
- ❑ 31
- ❑ 32
- ❑ 33
- ❑ 34
- ❑ 35
- ❑ 36
- ❑ 37
- ❑ 38
- ❑ 39
- ❑ 40
- ❑ 41
- ❑ 42
- ❑ 43
- ❑ 44
- ❑ 45
- ❑ 46
- ❑ 47

- ❑ 48
- ❑ 49
- ❑ 50
- ❑ More than 50
- ❑ Rather not say

At that time what type of sex did you have? (Please tick ALL that apply)

- ❑ Oral sex
- ❑ Vaginal sex
- ❑ Anal sex
- ❑ Mutual masturbation
- ❑ Rather not say
- ❑ Other

Please specify

____

When you had sex for the first time, were

- ❑ You and your partner equally willing
- ❑ You were more willing
- ❑ Your partner was more willing

When you had sex for the first time, if your partner was more willing would you say that:

- ❑ You were also willing
- ❑ You had to be persuaded by your partner
- ❑ You were forced

In the last 12 months, how many people have you had sex with?

- ❑ 1
- ❑ 2
- ❑ 3
- ❑ 4
- ❑ 5
- ❑ 6
- ❑ 7

- ❑ 8
- ❑ 9
- ❑ 10
- ❑ 11-20
- ❑ 21-30
- ❑ 31-40
- ❑ 41-50
- ❑ More than 50

Was this partner a:

- ❑ Man
- ❑ Woman

Was this a new partner (i.e. you had not had sex with them ever before)?

- ❑ Yes
- ❑ No

In the **last 12 months** how many of the {Q30} sex partner(s) were **MEN**?

- ❑ None
- ❑ 1
- ❑ 2
- ❑ 3
- ❑ 4
- ❑ 5
- ❑ 6
- ❑ 7
- ❑ 8
- ❑ 9
- ❑ 10
- ❑ 11-20
- ❑ 21-30
- ❑ 31-40
- ❑ 41-50
- ❑ More than 50

In the **last 12 months**, how many of the {Q30} sex partner(s) were **WOMEN**?
- ❑ None
- ❑ 1
- ❑ 2
- ❑ 3
- ❑ 4
- ❑ 5
- ❑ 6
- ❑ 7
- ❑ 8
- ❑ 9
- ❑ 10
- ❑ 11-20
- ❑ 21-30
- ❑ 31-40
- ❑ 41-50
- ❑ More than 50

Thinking about **all** the people you had sex with in the **last 12 months,** did any of them **overlap in time**? In other words did you have sex with someone (person A) then have sex with someone else (person B) then you had sex with person A again?
- ❑ Yes
- ❑ No
- ❑ Dont remember

In the **last 12 months**, how many of the {Q30} sex partner(s) were **NEW** (i.e. you had not had sex with them ever before)?
- ❑ None
- ❑ 1
- ❑ 2
- ❑ 3
- ❑ 4
- ❑ 5
- ❑ 6
- ❑ 7
- ❑ 8

- ❑ 9
- ❑ 10
- ❑ 11-20
- ❑ 21-30
- ❑ 31-40
- ❑ 41-50
- ❑ More than 50

Was your new partner a:

- ❑ Male
- ❑ Female

How many of these {Q36} were **NEW MALE** partners (i.e. you had not had sex with them ever before)?

- ❑ None
- ❑ 1
- ❑ 2
- ❑ 3
- ❑ 4
- ❑ 5
- ❑ 6
- ❑ 7
- ❑ 8
- ❑ 9
- ❑ 10
- ❑ 11-20
- ❑ 21-30
- ❑ 31-40
- ❑ 41-50
- ❑ More than 50

How many of these {Q36} were **NEW FEMALE** partners (i.e. you had not had sex with them ever before)?

- ❑ None
- ❑ 1
- ❑ 2
- ❑ 3
- ❑ 4
- ❑ 5
- ❑ 6
- ❑ 7

- ❑ 8
- ❑ 9
- ❑ 10
- ❑ 11-20
- ❑ 21-30
- ❑ 31-40
- ❑ 41-50
- ❑ More than 50

Have you or your partner used any of the following methods to avoid pregnancy in the **last 12 months**? (Tick **ALL** that apply)

- ❑ None / Not applicable
- ❑ The Pill
- ❑ Male condom
- ❑ Female condom
- ❑ Morning after pill
- ❑ Emergency intra-uterine device (IUD)
- ❑ Coil/intra-uterine device (IUD)
- ❑ Hormonal IUD – MIRENA
- ❑ Cap/diaphragm
- ❑ Injections
- ❑ Spermicides (foams/gels/sprays/pessaries)
- ❑ Natural family planning (safe period/rhythm method)
- ❑ Withdrawal
- ❑ Implants
- ❑ Sterilisation
- ❑ Vasectomy
- ❑ Abstinence
- ❑ Other method of protection

Please specify

In the **last 12 months**, how many times did you or your partner use emergency contraception (e.g. the ‘morning after pill’ or an emergency IUD)?

- ❑ Once
- ❑ 2-3 times
- ❑ 4-5 times
- ❑ 5-10 times
- ❑ More than 10 times
- ❑ Don't know

The following questions are about your sexual behaviour in the last 3 months

Have you had sex in the last 3 months?

- ❑ Yes
- ❑ No

How many sexual partners did you have in the **last 3 months**?

- ❑ 1
- ❑ 2
- ❑ 3
- ❑ 4
- ❑ 5
- ❑ 6
- ❑ 7
- ❑ 8
- ❑ 9
- ❑ 10
- ❑ 11-20
- ❑ 21-30
- ❑ 31-40
- ❑ 41-50
- ❑ More than 50

In the **last 3 months,** how many of these {Q43} were **men**?

- ❑ None
- ❑ 1
- ❑ 2
- ❑ 3
- ❑ 4
- ❑ 5
- ❑ 6
- ❑ 7
- ❑ 8
- ❑ 9
- ❑ 10
- ❑ 11-20
- ❑ 21-30
- ❑ 31-40
- ❑ 41-50
- ❑ More than 50

In the **last 3 months,** how many of these {Q43} were **women**?

- ❑ None
- ❑ 1
- ❑ 2
- ❑ 3
- ❑ 4
- ❑ 5
- ❑ 6
- ❑ 7
- ❑ 8
- ❑ 9
- ❑ 10
- ❑ 11-20
- ❑ 21-30
- ❑ 31-40
- ❑ 41-50
- ❑ More than 50

The following section is about your MOST RECENT sex partner in the last 3 months.

Think about the **LAST PERSON** you had sex with while answering the following questions.

When did you **most recently** have sex with this person?

- ❑ In the last 7 days
- ❑ 1-2 weeks ago
- ❑ More than 2 weeks ago but in the last month
- ❑ 1-3 months ago

When did you **first** have sex with this person?

- ❑ In the last 7 days
- ❑ 1-2 weeks ago
- ❑ More than 2 weeks ago but in the last month
- ❑ 1-3 months ago
- ❑ 4-6 months ago
- ❑ 7-12 months ago
- ❑ 1-5 years ago
- ❑ More than 5 years ago

Was your **last** sex partner:

- ❑ Male
- ❑ Female
- ❑ Other

Please specify

____

____

How old was your **last** sex partner? (If you don't know their exact age, give an estimate)

- ❑ Under 15
- ❑ 15
- ❑ 16
- ❑ 17
- ❑ 18
- ❑ 19
- ❑ 20
- ❑ 21
- ❑ 22
- ❑ 23
- ❑ 24
- ❑ 25
- ❑ 26
- ❑ 27
- ❑ 28
- ❑ 29
- ❑ 30
- ❑ 31
- ❑ 32
- ❑ 33
- ❑ 34
- ❑ 35
- ❑ 36
- ❑ 37
- ❑ 38
- ❑ 39
- ❑ 40
- ❑ 41

- ❑ 42
- ❑ 43
- ❑ 44
- ❑ 45
- ❑ 46
- ❑ 47
- ❑ 48
- ❑ 49
- ❑ 50
- ❑ 51
- ❑ 52
- ❑ 53
- ❑ 54
- ❑ 55
- ❑ 56
- ❑ 57
- ❑ 58
- ❑ 59
- ❑ 60
- ❑ 61
- ❑ 62
- ❑ 63
- ❑ 64
- ❑ 65
- ❑ 66
- ❑ 67
- ❑ 68
- ❑ 69
- ❑ 70
- ❑ 71
- ❑ 72
- ❑ 73
- ❑ 74
- ❑ 75
- ❑ 76
- ❑ 77
- ❑ 78
- ❑ 79

- ❑ 80
- ❑ Over 80

Which of the following best describes your **last** partner's ethnic group?

- ❑ White: English/Welsh/Scottish/Northern Irish/British
- ❑ White Irish
- ❑ Any other white background
- ❑ Black/ Black British: African
- ❑ Black/ Black British: Caribbean
- ❑ Black/ Black British: Any other Black background
- ❑ Asian/ Asian British: Indian
- ❑ Asian/Asian British: Pakistani
- ❑ Asian/Asian British: Bangladeshi
- ❑ Asian/Asian British: Chinese
- ❑ Asian/Asian British: Any other Asian background
- ❑ Mixed: White and Black Caribbean
- ❑ Mixed: White and Black African
- ❑ Mixed: White and Asian
- ❑ Mixed: any other mixed/multiple background
- ❑ Arab
- ❑ Any other ethnic group
- ❑ Decline to answer

Please specify

____

Please specify

____

Please specify

____

Please specify

____

Please specify

____

When you last had sex with this person, you:

- ❑ were (are) married to this person
- ❑ were (are) in a committed relationship, but you were (are) not married to this person
- ❑ were (are) not in a committed relationship but you have sex with this person regularly
- ❑ had recently met this person
- ❑ had just met this person for the 1st time

How did you meet your **last** sex partner? Choose only **one** option

- ❑ At school
- ❑ At college/university
- ❑ At work (or through work)
- ❑ In a pub, bar, night club, disco, or dance
- ❑ Introduced by friends or family
- ❑ Through sports club, faith group or other organisation
- ❑ On holiday or while travelling
- ❑ Internet dating website
- ❑ Other dating agency/personal ads
- ❑ Facebook
- ❑ Twitter
- ❑ Instagram
- ❑ Pandora
- ❑ WhatsApp
- ❑ Other social media websites
- ❑ Online but not through dating website or social media
- ❑ Had always known each other (eg as family friends)
- ❑ Neighbour/lived locally/flat share

- ❑ Arranged marriage
- ❑ In a public place (e.g. park, café, shop, public transport)
- ❑ Partner was a sex worker
- ❑ Partner was my client
- ❑ Other

Please specify

____

Please specify

____

Many people find it difficult to use condoms regularly. Did you use condom when you last had sex with your **last** sex partner?

- ❑ Yes
- ❑ No
- ❑ We only had oral sex

Why did you **not** use condoms the last time you had sex with your LAST partner? Tick **ALL** that apply

- ❑ We did not think about using a condom
- ❑ My partner does not like to use condoms
- ❑ I don't like to use condoms
- ❑ We both decided not to use condoms
- ❑ I asked my partner to use a condom but my partner refused
- ❑ I find it difficult to ask my partner to use a condom
- ❑ I / my partner does not mind getting pregnant
- ❑ We had used a condom but it broke / tore
- ❑ We had used a condom but it did not fit / slipped
- ❑ We did not have a condom
- ❑ I/ my partner was drunk
- ❑ I/my partner had taken recreational drugs (e.g. marijuana/weed)
- ❑ Other

Please specify

____

Did either you or your **last sex partner** use any (other) method to prevent pregnancy when you last had sex?

- ❑ Yes
- ❑ No
- ❑ Not applicable

What method was used to prevent pregnancy when you last had sex? Tick ALL that apply

- ❑ The Pill
- ❑ Male condom
- ❑ Female condom
- ❑ Morning after pill
- ❑ Emergency intra-uterine device (IUD)
- ❑ Coil/intra-uterine device (IUD)
- ❑ Hormonal IUD – MIRENA
- ❑ Cap/diaphragm
- ❑ Injections
- ❑ Spermicides (foams/gels/sprays/pessaries)
- ❑ Natural family planning (safe period/rhythm method)
- ❑ Withdrawal
- ❑ Implants
- ❑ Sterilisation
- ❑ Vasectomy
- ❑ Abstinence
- ❑ Other method of protection

Please specify

____
____

Do you expect to have sex with this person again?

- ❑ Yes
- ❑ Probably
- ❑ I don't know
- ❑ Probably not
- ❑ No

Now think about the 2nd last person you had sex with in the last 3 months (i.e. the person you had sex with BEFORE your last sex partner). These questions are the same as you have just answered but about your 2nd last partner:

Think about the **SECOND LAST PERSON** you had sex with while answering the following questions.

When did you **most recently** have sex with this person?
- ❑ In the last 7 days
- ❑ 1-2 weeks ago
- ❑ More than 2 weeks ago but in the last month
- ❑ 1-3 months ago

When did you **first** have sex with this person?
- ❑ In the last 7 days
- ❑ 1-2 weeks ago
- ❑ More than 2 weeks ago but in the last month
- ❑ 1-3 months ago
- ❑ 4-6 months ago
- ❑ 7-12 months ago
- ❑ 1-5 years ago
- ❑ More than 5 years ago

Was your **second last** sex partner:
- ❑ Male
- ❑ Female
- ❑ Other

Please specify

____

How old was your **second last** sex partner? (If you don't know their exact age, give an estimate)

- ❑ Under 15
- ❑ 15
- ❑ 16
- ❑ 17
- ❑ 18
- ❑ 19
- ❑ 20
- ❑ 21
- ❑ 22
- ❑ 23
- ❑ 24
- ❑ 25
- ❑ 26
- ❑ 27
- ❑ 28
- ❑ 29
- ❑ 30
- ❑ 31
- ❑ 32
- ❑ 33
- ❑ 34
- ❑ 35
- ❑ 36
- ❑ 37
- ❑ 38
- ❑ 39
- ❑ 40
- ❑ 41
- ❑ 42
- ❑ 43
- ❑ 44
- ❑ 45
- ❑ 46
- ❑ 47
- ❑ 48
- ❑ 49

- ❑ 50
- ❑ 51
- ❑ 52
- ❑ 53
- ❑ 54
- ❑ 55
- ❑ 56
- ❑ 57
- ❑ 58
- ❑ 59
- ❑ 60
- ❑ 61
- ❑ 62
- ❑ 63
- ❑ 64
- ❑ 65
- ❑ 66
- ❑ 67
- ❑ 68
- ❑ 69
- ❑ 70
- ❑ 71
- ❑ 72
- ❑ 73
- ❑ 74
- ❑ 75
- ❑ 76
- ❑ 77
- ❑ 78
- ❑ 79
- ❑ 80
- ❑ Over 80

Which of the following best describes your **second last** partner's ethnic group?

- ❑ White: English/Welsh/Scottish/Northern Irish/British
- ❑ White Irish
- ❑ Any other white background
- ❑ Black/ Black British: African
- ❑ Black/ Black British: Caribbean
- ❑ Black/ Black British: Any other Black background
- ❑ Asian/ Asian British: Indian
- ❑ Asian/Asian British: Pakistani
- ❑ Asian/Asian British: Bangladeshi
- ❑ Asian/Asian British: Chinese
- ❑ Asian/Asian British: Any other Asian background
- ❑ Mixed: White and Black Caribbean
- ❑ Mixed: White and Black African
- ❑ Mixed: White and Asian
- ❑ Mixed: any other mixed/multiple background
- ❑ Arab
- ❑ Any other ethnic group
- ❑ Decline to answer

Please specify

____

Please specify

____

Please specify

____

Please specify

____

Please specify

____

When you last had sex with your **second last** partner, you:

- ❑ were (are) married to this person
- ❑ were (are) in a committed relationship, but you were (are) not married to this person
- ❑ were (are) not in a committed relationship but you have sex with this person regularly
- ❑ had recently met this person
- ❑ had just met this person for the 1st time

How did you meet your **second last** sex partner? Choose only **one** option

- ❑ At school
- ❑ At college/university
- ❑ At work (or through work)
- ❑ In a pub, bar, night club, disco, or dance
- ❑ Introduced by friends or family
- ❑ Through sports club, faith group or other organisation
- ❑ On holiday or while travelling
- ❑ Internet dating website
- ❑ Other dating agency/personal ads
- ❑ Facebook
- ❑ Twitter
- ❑ Instagram
- ❑ Pandora
- ❑ WhatsApp

- ❑ Other social media websites
- ❑ Online but not through dating website or social media
- ❑ Had always known each other (eg as family friends)
- ❑ Neighbour/lived locally/flat share
- ❑ Arranged marriage
- ❑ In a public place (e.g. park, café, shop, public transport)
- ❑ Partner was a sex worker
- ❑ Partner was my client
- ❑ Other

Please specify

____

Please specify

____

Many people find it difficult to use condoms regularly. Did you use condom when you last had sex with your **second last** partner?

- ❑ Yes
- ❑ No
- ❑ We only had oral sex

Why did you **not** use condoms the last time you had sex with your **second last** partner? Tick **ALL** that apply

- ❑ We did not think about using a condom
- ❑ My partner does not like to use condoms
- ❑ I don't like to use condoms
- ❑ We both decided not to use condoms
- ❑ I asked my partner to use a condom but my partner refused
- ❑ I find it difficult to ask my partner to use a condom
- ❑ I / my partner does not mind getting pregnant
- ❑ We had used a condom but it broke / tore
- ❑ We had used a condom but it did not fit / slipped
- ❑ We did not have a condom
- ❑ I/ my partner was drunk

- ❑ I/my partner had taken recreational drugs (e.g. marijuana/weed)
- ❑ Other

Please specify

____

Did either you or your **second last** partner use any (other) method to prevent pregnancy when you last had sex?

- ❑ Yes
- ❑ No
- ❑ Not applicable

What method was used to prevent pregnancy when you last had sex with your **second last partner**? Tick **ALL** that apply

- ❑ The Pill
- ❑ Male condom
- ❑ Female condom
- ❑ Morning after pill
- ❑ Emergency intra-uterine device (IUD)
- ❑ Coil/intra-uterine device (IUD)
- ❑ Hormonal IUD – MIRENA
- ❑ Cap/diaphragm
- ❑ Injections
- ❑ Spermicides (foams/gels/sprays/pessaries)
- ❑ Natural family planning (safe period/rhythm method)
- ❑ Withdrawal
- ❑ Implants
- ❑ Sterilisation
- ❑ Vasectomy
- ❑ Abstinence
- ❑ Other method of protection

Please specify

____

Do you expect to have sex with your **second last** partner again?
- ❑ Yes
- ❑ Probably
- ❑ I dont know
- ❑ Probably not
- ❑ No

Now think about the THIRD LAST person you had sex with. <u>This is the last partner we will ask you about</u>.

Think about the **THIRD LAST PERSON** you had sex with while answering the following questions.

When did you **most recently** have sex with this person?
- ❑ in the last 7 days
- ❑ 1-2 weeks ago
- ❑ More than 2 weeks ago but in the last month
- ❑ 1-3 months ago

When did you **first** have sex with this person?
- ❑ in the last 7 days
- ❑ 1-2 weeks ago
- ❑ More than 2 weeks ago but in the last month
- ❑ 1-3 months ago
- ❑ 4-6 months ago
- ❑ 7-12 months ago
- ❑ 1-5 years ago
- ❑ More than 5 years ago

Was your **third last** sex partner:

- ❑ Male
- ❑ Female
- ❑ Other

Please specify

____

____

How old was your **third last** sex partner? (If you don’t know their exact age, give an estimate)

- ❑ Under 15
- ❑ 15
- ❑ 16
- ❑ 17
- ❑ 18
- ❑ 19
- ❑ 20
- ❑ 21
- ❑ 22
- ❑ 23
- ❑ 24
- ❑ 25
- ❑ 26
- ❑ 27
- ❑ 28
- ❑ 29
- ❑ 30
- ❑ 31
- ❑ 32
- ❑ 33
- ❑ 34
- ❑ 35
- ❑ 36
- ❑ 37
- ❑ 38
- ❑ 39
- ❑ 40
- ❑ 41
- ❑ 42

- ❑ 43
- ❑ 44
- ❑ 45
- ❑ 46
- ❑ 47
- ❑ 48
- ❑ 49
- ❑ 50
- ❑ 51
- ❑ 52
- ❑ 53
- ❑ 54
- ❑ 55
- ❑ 56
- ❑ 57
- ❑ 58
- ❑ 59
- ❑ 60
- ❑ 61
- ❑ 62
- ❑ 63
- ❑ 64
- ❑ 65
- ❑ 66
- ❑ 67
- ❑ 68
- ❑ 69
- ❑ 70
- ❑ 71
- ❑ 72
- ❑ 73
- ❑ 74
- ❑ 75
- ❑ 76
- ❑ 77
- ❑ 78
- ❑ 79
- ❑ 80
- ❑ Over 80

Which of the following best describes your **third last** partner's ethnic group?

- ❑ White: English/Welsh/Scottish/Northern Irish/British
- ❑ White Irish
- ❑ Any other white background
- ❑ Black/ Black British: African
- ❑ Black/ Black British: Caribbean
- ❑ Black/ Black British:Any other Black background
- ❑ Asian/ Asian British: Indian
- ❑ Asian/Asian British: Pakistani
- ❑ Asian/Asian British: Bangladeshi
- ❑ Asian/Asian British: Chinese
- ❑ Asian/Asian British: Any other Asian background
- ❑ Mixed: White and Black Caribbean
- ❑ Mixed: White and Black African
- ❑ Mixed: White and Asian
- ❑ Mixed: any other mixed/multiple background
- ❑ Arab
- ❑ Any other ethnic group
- ❑ Decline to answer

Please specify

____

Please specify

____

Please specify

____

Please specify

____

Please specify

____

When you last had sex with this person, you:

- ❑ were (are) married to this person
- ❑ were (are) in a committed relationship, but you were (are) not married to this person
- ❑ were (are) not in a committed relationship but you have sex with this person regularly
- ❑ had recently met this person
- ❑ had just met this person for the 1st time

How did you meet your **third last** sex partner? Choose only **one** option

- ❑ At school
- ❑ At college/university
- ❑ At work (or through work)
- ❑ In a pub, bar, night club, disco, or dance
- ❑ Introduced by friends or family
- ❑ Through sports club, faith group or other organisation
- ❑ On holiday or while travelling
- ❑ Internet dating website
- ❑ Other dating agency/personal ads
- ❑ Facebook
- ❑ Twitter
- ❑ Instagram
- ❑ Pandora
- ❑ WhatsApp
- ❑ Other social media websites
- ❑ Online but not through dating website or social media
- ❑ Had always known each other (eg as family friends)
- ❑ Neighbour/lived locally/flat share
- ❑ Arranged marriage
- ❑ In a public place (e.g. park, café, shop, public transport)
- ❑ Partner was a sex worker
- ❑ Partner was my client
- ❑ Other

Please specify

____

____

Please specify

____

Many people find it difficult to use condoms regularly. Did you use condom when you last had sex with your **third last** sex partner?

- ❑ Yes
- ❑ No
- ❑ We only had oral sex

Why did you **not** use condoms the last time you had sex with your **third last** partner? Tick **ALL** that apply

- ❑ We did not think about using a condom
- ❑ My partner does not like to use condoms
- ❑ I don't like to use condoms
- ❑ We both decided not to use condoms
- ❑ I asked my partner to use a condom but my partner refused
- ❑ I find it difficult to ask my partner to use a condom
- ❑ I / my partner does not mind getting pregnant
- ❑ We had used a condom but it broke / tore
- ❑ We had used a condom but it did not fit / slipped
- ❑ We did not have a condom
- ❑ I/ my partner was drunk
- ❑ I/my partner had taken recreational drugs (e.g. marijuana/weed)
- ❑ Other

Please specify

____

Did either you or your **third last** sex partner use any (other) method to prevent pregnancy when you last had sex?

- ❑ Yes
- ❑ No
- ❑ Not applicable

What method was used to prevent pregnancy when you last had sex with your **third last partner**? Tick **ALL** that apply

- ❑ The Pill
- ❑ Male condom
- ❑ Female condom
- ❑ Morning after pill
- ❑ Emergency intra-uterine device (IUD)
- ❑ Coil/intra-uterine device (IUD)
- ❑ Hormonal IUD – MIRENA
- ❑ Cap/diaphragm
- ❑ Injections
- ❑ Spermicides (foams/gels/sprays/pessaries)
- ❑ Natural family planning (safe period/rhythm method)
- ❑ Withdrawal
- ❑ Implants
- ❑ Sterilisation
- ❑ Vasectomy
- ❑ Abstinence
- ❑ Other method of protection

Please specify

____

Do you expect to have sex with this person again?

- ❑ Yes
- ❑ Probably
- ❑ I don't know
- ❑ Probably not
- ❑ No

We would like to know your opinion about having more than one sex partner IN THE SAME TIME PERIOD. By this we mean person X has sex with person A then with person B, then has sex with person A again.

Please tick your response to EACH of the following statements:

	Strongly disagree	Disagree	Slightly disagree	Slightly agree	Agree	Strongly agree
It is in my nature to have more than one sex partner in the same time period.	❑	❑	❑	❑	❑	❑
I respect men who have multiple sex partners	❑	❑	❑	❑	❑	❑
I need to have sex with more than one partner for variety.	❑	❑	❑	❑	❑	❑
It is OK to have sex with my child's mother/ father even if I am in a relationship with someone else.	❑	❑	❑	❑	❑	❑
I would be respected if I had multiple sex partners	❑	❑	❑	❑	❑	❑
Being in a relationship with just one person is boring.	❑	❑	❑	❑	❑	❑
Having multiple sex partner in the same time period makes me feel like a man/woman.	❑	❑	❑	❑	❑	❑
It is smart to have outside sex partners in the same time period as a "back up" (in case things do not work out with my main partner).	❑	❑	❑	❑	❑	❑
One sex partner in the same time period cannot meet all my needs.	❑	❑	❑	❑	❑	❑
I cannot control my sexual impulses.	❑	❑	❑	❑	❑	❑

The following questions ask about your sexual health and use of sexual health services.

Why did you come to the clinic when you were offered this survey? (Tick ALL that apply)

- ❑ I have (or had) symptoms (e.g. itching, discharge etc)
- ❑ My symptoms didn’t go away since I last came here for treatment
- ❑ My partner has (or had) symptoms
- ❑ My partner had been diagnosed with an STI so I came to the clinic to test
- ❑ Someone from the clinic contacted me and asked me to come to the clinic
- ❑ I did not have symptoms but wanted a check-up
- ❑ I wanted a HIV test
- ❑ My GP/practice nurse told me to come here
- ❑ Other

Please specify

____

____

How long ago did your symptoms start?

- ❑ In the last 7 days
- ❑ 1-2 weeks ago
- ❑ More than 2 weeks ago but in the last month
- ❑ More than one month ago but in the last 3 months
- ❑ More than 3 months ago but in the last 12 months
- ❑ More than a year ago

Before coming here today, did you try to get treatment or advice for your symptoms from anywhere else?

- ❑ Yes
- ❑ No

Since your symptoms started, have you had sex?

- ❑ No
- ❑ Yes, only with 1 partner
- ❑ Yes, with more than 1 partner

When was the **last time** you were diagnosed/treated for a STI?
- ❑ In the last 7 days
- ❑ 1-2 weeks ago
- ❑ More than 2 weeks ago but in the last month
- ❑ More than one month ago but in the last 3 months
- ❑ More than 3 months ago but in the last 12 months
- ❑ More than a year ago
- ❑ Never been diagnosed/treated for a STI

At that time what were you diagnosed with/treated for? Tick ALL that apply.
- ❑ Chlamydia
- ❑ Gonorrhoea
- ❑ Genital Warts (venereal warts)
- ❑ Syphilis
- ❑ Trichomonas vaginalis (Trich, TV)
- ❑ Herpes (genital herpes)
- ❑ Hepatitis B
- ❑ NSU (Non Specific Urethritis), NGU (Non Gonococcal Urethritis)
- ❑ Epididymitis
- ❑ HIV
- ❑ Can't remember

At that time did the clinic staff advise you to inform your sexual partners to test for STIs /come to clinic?
- ❑ Yes
- ❑ No
- ❑ Can't remember

At that time did you inform your sexual partner that **you had been diagnosed with STIs/ treated for STIs**?
- ❑ Yes
- ❑ No
- ❑ Can't remember

At that time, did you inform your sexual partners to **test/take treatment for STIs**?

- ❑ Yes, I told ALL my partners
- ❑ Yes, I told SOME of my partners
- ❑ No, I didn’t tell any partners
- ❑ Can't remember

At that time, how did you inform your sexual partners to test for STIs/come to clinic? (Please tick ALL that apply)

- ❑ In person
- ❑ Via a text message
- ❑ Via an email
- ❑ Via telephone
- ❑ Via social media
- ❑ Via a friend
- ❑ Via a clinic health advisor/clinic staff
- ❑ Other

Please specify

____

____

At that time, HOW MANY sexual partners did you inform to test for the infection/come to the clinic?

____

At that time, why did you not inform (some of) your sexual partners to test for the infection/come to the clinic? (Tick ALL the apply)

- ❑ My partner(s) lives outside the UK
- ❑ I was embarrassed to tell my partner(s) about the infection
- ❑ I was scared of telling my partner(s) about the infection
- ❑ I was worried that my partner(s) would leave me
- ❑ I did not have contact details of my partner(s)
- ❑ I was not too concerned about telling my main partner
- ❑ I was not too concerned about telling my casual/one-off partners
- ❑ Other

Please specify

____

Thank you for your interest in the survey but your answers show that you do not qualify to take part in this survey. Now please press SUBMIT/TICK to exit.

Thank you for letting us know that you do not wish to participate in the survey. Now press next Submit / Tick button to exit the survey.

Thank you for completing the survey, the clinic staff will NOT see your responses to this survey, but you can discuss any issues raised from completing this survey if you wish to do so. Now please press Submit/Tick button to exit the survey.
